# *Caenorhabditis elegans* Histone Deacetylase *hda-1* Is Required for Morphogenesis of the Vulva and LIN-12/Notch-Mediated Specification of Uterine Cell Fates

**DOI:** 10.1534/g3.113.006999

**Published:** 2013-08-01

**Authors:** Ayush Vasant Ranawade, Philip Cumbo, Bhagwati P. Gupta

**Affiliations:** Department of Biology, McMaster University, Hamilton, ON L8S 4K1 Canada

**Keywords:** *C. elegans*, morphogenesis, reproductive system, histone deacetylase, *hda-1*

## Abstract

Chromatin modification genes play crucial roles in development and disease. In *Caenorhabditis elegans*, the class I histone deacetylase family member *hda-1*, a component of the nucleosome remodeling and deacetylation complex, has been shown to control cell proliferation. We recovered *hda-1* in an RNA interference screen for genes involved in the morphogenesis of the egg-laying system. We found that *hda-1* mutants have abnormal vulva morphology and vulval-uterine connections (*i.e.*, no uterine-seam cell). We characterized the vulval defects by using cell fate-specific markers and found that *hda-1* is necessary for the specification of all seven vulval cell types. The analysis of the vulval-uterine connection defect revealed that *hda-1* is required for the differentiation of the gonadal anchor cell (AC), which in turn induces ventral uterine granddaughters to adopt π fates, leading to the formation of the uterine-seam cell. Consistent with these results, *hda-1* is expressed in the vulva and AC. A search for *hda-1* target genes revealed that *fos-1* (*fos* proto-oncogene family) acts downstream of *hda-1* in vulval cells, whereas *egl-43* (*evi1* proto-oncogene family) and *nhr-67* (tailless homolog, NHR family) mediate *hda-1* function in the AC. Furthermore, we showed that AC expression of *hda-1* plays a crucial role in the regulation of the *lin-12/Notch* ligand *lag-2* to specify π cell fates. These results demonstrate the pivotal role of *hda-1* in the formation of the vulva and the vulval-uterine connection. Given that *hda-1* homologs are conserved across the phyla, our findings are likely to provide a better understanding of HDAC1 function in development and disease.

The formation of tissues and organs involves a complex series of cellular events such as cell proliferation, differentiation, and migration. These processes are orchestrated by a large number of genes, including transcription factors, signaling molecules, and chromatin modifiers. Chromatin-modifying proteins regulate transcription by inducing changes in chromatin structure that affect the accessibility of regulatory DNA sequences to the transcriptional machinery. These regulatory proteins have been identified in many organisms ranging from yeast to humans and are known to form complexes (*e.g.*, NURD/CoREST) with distinct regulatory modes and functions.

The NURD chromatin complex is unique in that it combines the activity of both histone modifiers (histone deacetylases, or HDACs) and chromatin remodelers (Mi-2 ATPase) into one complex. The HDACs deacetylate histone tails, leading to chromatin compaction, whereas the Mi-2 ATPase disrupts the binding of histones to DNA, which allows transcription factors to have easier access to the DNA to control gene expression (Xue *et al.* 1998). The activity of HDACs is counteracted by another group of enzymes, histone acetyltransferases, that acetylate histone tails and make chromatin more accessible to transcriptional machinery. The balance between HDAC and histone acetyltransferase activity ensures precise control of gene expression, and failure to regulate their activity can cause cancers and metastatic growth. For example, many HDACs are highly expressed in lymphomas of both classical Hodgkin and non-Hodgkin types (Gloghini *et al.* 2009). HDAC inhibitors have emerged as a powerful new class of small-molecule therapeutics that acts through the regulation of the acetylation states of histone proteins (a form of epigenetic modulation) and other nonhistone protein targets. Although HDAC inhibitors have been successfully implemented as therapeutics, the mechanistic details of how these proteins interact with other cellular machinery and signaling pathways during normal development and disease are poorly understood.

The egg-laying system of *Caenorhabditis elegans* offers many advantages for the study of how chromatin remodelers and histone modifiers regulate gene expression to control tissue morphogenesis. The vulva, a passageway for laying eggs, is formed by 22 cells that arise from successive divisions of three vulval precursor cells (VPCs): P5.p, P6.p, and P7.p. The VPCs are induced by evolutionarily conserved signaling pathways mediated by LET-60/Ras, LIN-12/Notch, and Wnt. The Ras pathway induces a 1° fate in P6.p through an EGF-secreted signal from the overlying anchor cell (AC). This in turn activates the LIN-12/Notch pathway from the P6.p cell in a lateral manner, inducing a 2° fate in both P5.p and P7.p (Greenwald 2005; Sternberg 2005). The Wnt pathway is also involved in 2° fate specification and appears to act in parallel and through crosstalk with the LIN-12/Notch pathway (Seetharaman *et al.* 2010). In addition to signaling pathway components, genetic screens in *C. elegans* have also identified a number of genes known as SynMuv (synthetic multivulva) genes, a gene family that interacts with the Ras pathway to negatively regulate vulval cell proliferation (Cui *et al.* 2006; Cui and Han 2007). SynMuv genes are divided into three different classes (A, B, and C) based on their genetic properties, such that mutations in any one of the classes do not (or rarely) affect the VPC induction pattern, but in combination with the other classes, give rise to a multivulva (Muv) phenotype (Fay and Yochem 2007). Genetic and biochemical studies have shown that class B SynMuv genes encode components of chromatin remodeling complexes, such as *let-418/Mi2* and *hda-1/hdac1* (Fay and Yochem 2007).

Nucleosome remodeling and deacetylation (NURD) complex proteins in *C. elegans* play important roles during development. HDA-1 (HDAC1), a catalytic subunit of NURD, is required for embryogenesis, gonadogenesis, germ cell formation, neuronal axon guidance, and vulval development (Dufourcq *et al.* 2002; Zinovyeva *et al.* 2006). In the vulva, *hda-1* knockdown has been shown to cause a weak Muv phenotype in combination with mutations in any one of the class A and class B SynMuv genes (Lu and Horvitz 1998; Solari and Ahringer 2000). Subsequently, a similar phenotype was reported in *hda-1* mutants alone (Dufourcq *et al.* 2002; Zinovyeva *et al.* 2006), although the SynMuv interaction was not observed (Dufourcq *et al.* 2002). In addition, vulval cells in *hda-1* animals fail to migrate and form ectopic invaginations (Dufourcq *et al.* 2002). It is unclear whether the invagination defect is another factor contributing to the Muv phenotype because VPC induction patterns were not examined.

We performed an RNA interference (RNAi) screen to identify the transcription and chromatin-associated factors involved in vulva and vulva−uterine connection formation. The screen identified new genes as well as previously discovered genes, including *hda-1*. In this study, we investigated the role of *hda-1* in detail. The vulval morphology defect in *hda-1* animals suggests that *hda-1* is involved in cell differentiation and cell migration processes. Furthermore, *hda-1* is expressed in vulval cells in a temporally restricted manner. To understand how *hda-1* controls vulval development, we searched for interacting genes and found that the *fos* proto-oncogene family member *fos-1b* and the LIM-Hox family member *lin-11* act genetically downstream of *hda-1* in vulval cells.

In addition to vulva development, we found that *hda-1* is also involved in the formation of the vulval−uterine connection. In *hda-1* mutants the uterine seam cell (utse) fails to form due to defect in π cell fates, as determined by expression analysis of 2 important π lineage-specific transcription factors, *lin-11* and *egl-13* (SOX family). Further analysis of the role of *hda-1* in π cell fate specification revealed that *hda-1* acts in the AC to signal ventral uterine (VU) granddaughters to adopt π fates. This process involves *egl-43* (*evi1* proto-oncogene family) and *nhr-67* (*tailless* ortholog of NHR family)-mediated regulation of *lag-2* (DSL ligand) expression, which in turn activates *lin-12/Notch* signaling in VU granddaughters. Taken together, our findings establish *hda-1* as a key regulator of vulva and uterine cell morphogenesis.

## Materials and Methods

### Strains and general methods

All strains were maintained at 20°. Worm cultures and genetic manipulations were conducted as described previously (Brenner 1974). The mutations and transgene markers used in this study are listed below. The linkage group is indicated when known.

N2 (wild type), *arEx1352[lag-2*::*gfp + pha-4(+)]*, *ayIs4[egl-17*::*gfp + dpy-20(+)] I*, *bhEx53[pGLC9(daf-6*::*yfp) + unc-119(+)]*, *bhEx68[pGLC43(Cbr-hda-1*::*gfp) + unc-119(+)]*, *bhEx72[pGLC44(hda-1*::*gfp) + unc-119(+)]*, *deIs4[ajm-1*::*gfp + lin-39*::*gfp (yeast DNA) + dpy-20(+)] I*, *fos-1(ar105) V*, *hda-1(cw2) V*, *hda-1(e1795) V*, *inIs181*; *inIs182[ida-1*::*gfp]*, *kuIs29[pWH17(egl-13*::*gfp) + unc-119(+)] V*, *nIs408 [lin-29p*::*lin-29*::*mCherry + ttx-3p*::*gfp]*, *qIs56 [lag-2*::*gfp (pJK590) + unc-119(+)]V*, *qyIs174 [hlh-2p*::*gfp*::*hlh-2 + unc-119(+)]*, *sEx13706[rCes C53A5.3*::*gfp + pCeh361]*, *syIs49[zmp-1*::*gfp + dpy-20(+)] IV*, *stIs11476 [nhr-67*::H1-wCherry *+ unc-119(+)]*, *syls50[cdh-3*::*gfp + unc-119(+)] X*, *syIs54[ceh-2*::*gfp + unc-119(+)] II*, *syIs80[pPGF11.13(lin-11*::*gfp) + unc-119(+)] III*, *syIs123[fos-1a*::*yfp-TL + unc-119(+)] X*, *syIs137[fos-1b*::*cfp-TX + unc-119(+)] III*, *unc-119(ed4) III*, *zhEx216.2[egl-43-1.7-lp*::*gfp + unc-119(+)]*.

### Phenotypic analysis

The vulva and utse phenotypes were examined during the L3 and L4 stages. P(5−7).p cells divide between mid-L3 and early-L4 to generate a total of 22 progeny. The vulval toroids were visualized in mid-L4 animals using *ajm-1*::*gfp*. The π cells (on either side of the AC) and their progeny (immediately dorsal to the vulval tissue) were observed during the late-L3 and early to mid-L4 stages. The utse was detected as a thin membrane (hymen) in mid-L4 animals. The expression of *lag-2*::*gfp* was quantified in early to mid-L3 stage animals. Worms were scored for *egl-43*::*gfp*, *nhr-67*::*wcherry*, *hlh-2*::*gfp* and *lin-29*::*wcherry* expression at the mid-L3 stage.

We looked at four independently isolated stable lines for *hda-1*::*gfp* and 3 for *daf-6*::*yfp*. All strains showed identical pattern of expression.

We used multiple criteria to ensure that animals were examined at correct stages. The staging was based primarily on gonad morphology (Hall and Altun 2008). Because gonad morphology is defective in *hda-1* mutants, the appropriate stage was selected based on developmental timing of control animals. For π cell lineage analysis, we relied on *egl-13* and *lin-11* markers that show expression in π cells starting mid to late-L3 stage. For examination of π progeny and vulval cells we picked animals at L4 lethargus stage.

### Molecular biology and transgenics

The sequences of primers used in this study are as follows (5′ to 3′ orientation). The restriction enzyme sites are underlined.

GL176: TTTCTGCAGCCTTTCTGAAACCGGTTGTTTATTC,
GL177: GCAGGTACCACTAGAGGTTCAATTTGCAGAATCTGC,
GL354: CTCCCTTGACAGTTTCGGCAGTCCATTTC,
GL355 TCTCTGCAGTTCGAGTTCATTGTTGCCTG,
GL360: GATTGAATGCATGTTTGATGGTCGCAGTAGACTG,
GL363: ATCAAGCTTGTGCGTGCTCGCGGTTGTG.

The *hda-1*::*gfp* plasmids, pGLC44 (*C. elegans*) and pGLC43 (*Caenorhabditis briggsae*), were made by subcloning an *Nsi*I/*Hind*III-digested 1024-bp DNA fragment from *C. elegans* into the Fire lab vector pPD95.69 and an *Nsi*I/*Pst*I-digested 1030-bp DNA fragment from *C. briggsae* into pPD95.67 (polymerase chain reaction [PCR] primers GL363/GL360 and GL354/GL355, respectively). Because the *C. elegans* fragment contains all but approximately 250 bp of the DNA region between *hda-1* and its upstream gene, *ril-1*, it is formally possible that *gfp* expression in the transgenic animals is regulated by both the *hda-1* and *ril-1* enhancers. However, this is unlikely because *ril-1* has no known function in vulva and uterine cells (Hansen *et al.* 2005; Lemire *et al.* 2009). To construct *daf-6*::*yfp* (pGLC9), we inserted a 3-kb *Pst*I/*Kpn*I-digested 5′ regulatory DNA fragment (amplified by PCR using primers GL176/GL177) into pPD136.61.

Transgenic strains were generated by microinjection (Mello *et al.* 1991). In all cases *unc-119* was used as a rescue marker. The *hda-1*::*gfp* transgenic lines are *bhEx68*, *bhEx69*, *bhEx71* (all containing *C. briggsae hda-1*), and *bhEx72* (containing *C. elegans hda-1*). The *daf-6*::*yfp* lines are *bhEx53*, *bhEx54* and *bhEx55*.

### RNAi

RNAi experiments were performed using the Ahringer lab bacterial feeding library (Sanger Institute). The protocol has been described previously (Seetharaman *et al.* 2010). All experiments were repeated at least three times, and batches with similar results were pooled and analyzed. The RNAi phenotypes were compared with published results such as Pvl, defective vulval invagination, and sterility (www.wormbase.org) to ensure quality. The empty vector L4440-containing bacteria served as a negative control. Except where noted, feeding RNAi was performed in L1 larvae, which were synchronized as follows: gravid adults grown at 20° were treated with a hypochlorite solution for 4–5 min. Embryos were washed five times with M9 and then allowed to hatch in M9 for 16–30 hr at 20° with gentle agitation. The L1 worms were placed on feeding RNAi plates and maintained at 20°. The cells were plated on RNAi media plates and allowed to grow overnight before the plates were seeded with L1 worms. For double RNAi experiments, bacterial cultures of *hda-1*, *nhr-67*, *lin-29*, and *hlh-2* were mixed in equal proportion as described earlier (Penigault and Felix 2011). In these cases we examined batches in which animals exhibited phenotypes characteristic of both genes.

### Microscopy

Worms were mounted on agar pads as described previously (Wood 1988). L4 and young adults were examined under Nomarski optics using a Zeiss Axioimager D1 and a Nikon Eclipse 80i. For GFP reporter-expressing animals, epifluorescence was visualized by a Zeiss Axioimager D1 microscope equipped with the GFP filter HQ485LP (Chroma Technology). Confocal images were captured on a Leica DMI 6000B laser scanning microscope using Leica Application Suite Advanced software. All images were processed using NIH Image J (http://rsb.info.nih.gov/ij) and Illustrator and Photoshop (Adobe Inc.) software.

### Analysis of fluorescent reporters

Images of *gfp*-expressing animals were captured at the subsaturation level by optimizing the exposure time and gain. Green fluorescent protein (GFP) fluorescence in AC was quantified using ImageJ as described earlier (Schindler and Sherwood 2011). To summarize, AC was manually cropped, and the mean pixel intensity was measured (area of AC × mean pixel intensity in that area) after subtracting the background, and the data were plotted as a percentage of fluorescence intensity. For *lag-2*::*gfp* expression analysis, two different transgenic lines, *qIs56* and *arEx1352*, were used. In all cases only worms with expression in DTC were selected for analysis. Because *hda-1* was earlier shown to act as a class B synMuv gene and class B genes affect transgene expression levels (Hsieh *et al.* 1999; Wang *et al.* 2005), *hda-1* knockdown may cause transgene silencing globally. However, this possibility is less likely because *hda-1* mostly represses transcription (Whetstine *et al.* 2005). Also, Dufourcq *et al.* (2002) did not find global transcriptional silencing in *hda-1* mutants. In our case, we looked at the expression of marker genes in different tissues. Although the expression was reduced or eliminated in vulva or uterine cells, no obvious change in other tissues was observed.

### Data analysis

Statistical analyses were performed using InStat 2.0 (GraphPad Software Inc.) software. Two-tailed *P* values were calculated in unpaired Wilcoxon/Mann-Whitney tests and values less than 0.05 were considered to be statistically significant.

## Results

### RNAi screen for genes involved in vulva and vulva−uterine connection formation

**Figure 1:**
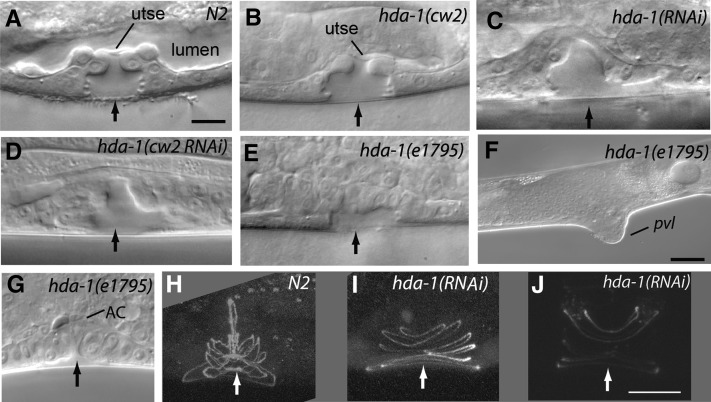
Vulval morphology in wild-type and *hda-1* mutant animals. Arrows mark the center of vulval invagination. (A) The wild-type L4 stage vulva has a characteristic invagination pattern. Compared with the wild type, the vulval morphology is defective in *hda*-1 mutant animals. (B) *hda-1(cw2)*, (C) *hda-1(RNAi)*, and (D) *hda-1(cw2)* treated with *hda-1* RNAi and (E) *hda-1(e1795)*. (F) Protruding vulva phenotype in adult *hda-1(e1795)* hermaphrodite. (G) The AC has failed to migrate in this animal. (H-J) *ajm-1*::*gfp* reveals fainter expression and wider vulval rings in *hda-1(RNAi)* animal compared with the wild type. (A−E, G) Scale bar is 10 μm; (F) scale bar is 30 μm; (H−J) scale bar is 50 μm.

We conducted a systematic RNAi screen for a subset of conserved transcription factors and genes involved in chromatin modification (Cui and Han 2007; Haerty *et al.* 2008). We fed age-synchronized N2 wild-type, L1-staged animals with dsRNA-expressing bacteria and examined the animals for abnormal vulval invagination in the L4 stage, and later, for protruding vulva (Pvl) phenotypes in adults. Of the 171 genes tested, RNAi-mediated knockdown of 34 different genes (20%) caused Pvl and/or vulva rupture defects, as observed under a dissecting microscope; this result was further confirmed by Nomarski microscopy. Vulval morphology was also defective in 16 of 34 of the knockdown strains (Figure 1, Supporting Information, Figure S1, and Table S1). One of these genes, the class I histone deacetylase family member *hda-1*, is a known negative regulator of vulval cell proliferation (Dufourcq *et al.* 2002; Lu and Horvitz 1998; Solari and Ahringer 2000).

### *hda-1* mutants exhibit abnormal vulva and vulval−uterine connections

The *hda-1(RNAi)* animals have a Pvl phenotype similar to that observed in two viable *hda-1* hypomorphs, *cw2* and *e1795* (Dufourcq *et al.* 2002; Zinovyeva *et al.* 2006). Upon careful examination we found that the Pvl penetrance is high in RNAi and *e1795* animals but very low in *cw2* (Table 1). Earlier, more than half of *cw2* animals (62%) were reported to be Pvl (Zinovyeva *et al.* 2006). This difference may be caused by the way Pvl phenotype was scored. In our case we counted only those protrusions that were big and clearly noticeable (see Figure 1F as an example). In addition to the Pvl defect, *hda-1* animals also showed abnormal morphology of the developing vulva. Specifically, vulval cells in L4 stage frequently failed to invaginate and that the vulva lacked the two mirror-symmetric halves characteristic of wild-type animals (compare Figure 1A with Figure 1, B−E). The defect was most severe in *hda-1(e1795)*, followed by *hda-1(RNAi)* and *hda-1(cw2)*. The *hda-1(cw2)* phenotype could be further enhanced by RNAi knockdown of *hda-1* (Figure 1D, Table 1), which is consistent with *cw2* being a hypomorphic allele.

**Table 1:** Vulval invagination and morphology defects in various genetic backgrounds

Genotype	Abnormal Invagination (L4 Stage)	Pvl (Adult)
*N2*	None (n > 100)	None (n > 100)
*hda-1(RNAi)*	72% (n = 190)	79% (n = 36)
*hda-1(e1795)*	100% (n = 43)	100% (n = 30)
*hda-1(cw2)*	68% (n = 45)	1.4% (n = 152)
*hda-1(cw2 RNAi)*	100% (n = 14)	100% (n = 30)

ND, not done; n, number of animals examined.

During the L4 stage, vulval cells migrate toward the center and invaginate to occupy stereotypic positions. Similar cell types subsequently fuse, generating toroidal rings that line the vulval cavity. We examined the possibility that abnormal vulval invagination in *hda-1*(RNAi) animals is caused by improper cell fusion events. To this end, we used an adherens junction marker, *ajm-1*::*gfp*, to visualize cell boundaries and vulval toroids (Sharma-Kishore *et al.* 1999). In wild-type L4 animals, *ajm-1*::*gfp* is expressed in seven concentric toroidal rings (vulA to vulF), each corresponding with the boundary between two different cell types (Figure 1H). We found that in the 60% (n = 25) *hda-1(RNAi)* animals, the vulval rings were defective. Specifically, the toroids were 40% (n = 5) wider than normal (*N2*, n = 2) and disorganized, and in some cases, had fewer than seven rings (Figure 1, I and J). These phenotypes may arise from abnormal morphogenetic movements and altered cell fates (see next section).

In addition to the vulva abnormalities, we also observed defects in the vulval-uterine connection in the *hda-1* animals. In the wild-type animals, a thin membrane consisting of a uterine seam cell (utse) is visible at the apex of the vulva (Figure 1A), whereas in the *hda-1(RNAi)* animals the membrane could not be clearly observed (Figure 1C). The morphology was only slightly abnormal in *hda-1(cw2)* animals (Figure 1B) but was clearly defective in *hda-1(cw2 RNAi)* and *hda-1(e1795)* animals (Figure 1, D and E). It is unclear whether the utse was absent altogether or was present but could not be identified due to an abnormal morphology. The uterine lumen was also frequently absent (Figure 1, C−E). In some cases, the AC failed to migrate and appeared to be located at the top of the vulval apex (Figure 1G).

### Vulval cells fail to differentiate in *hda-1* animals

**Figure 2:**
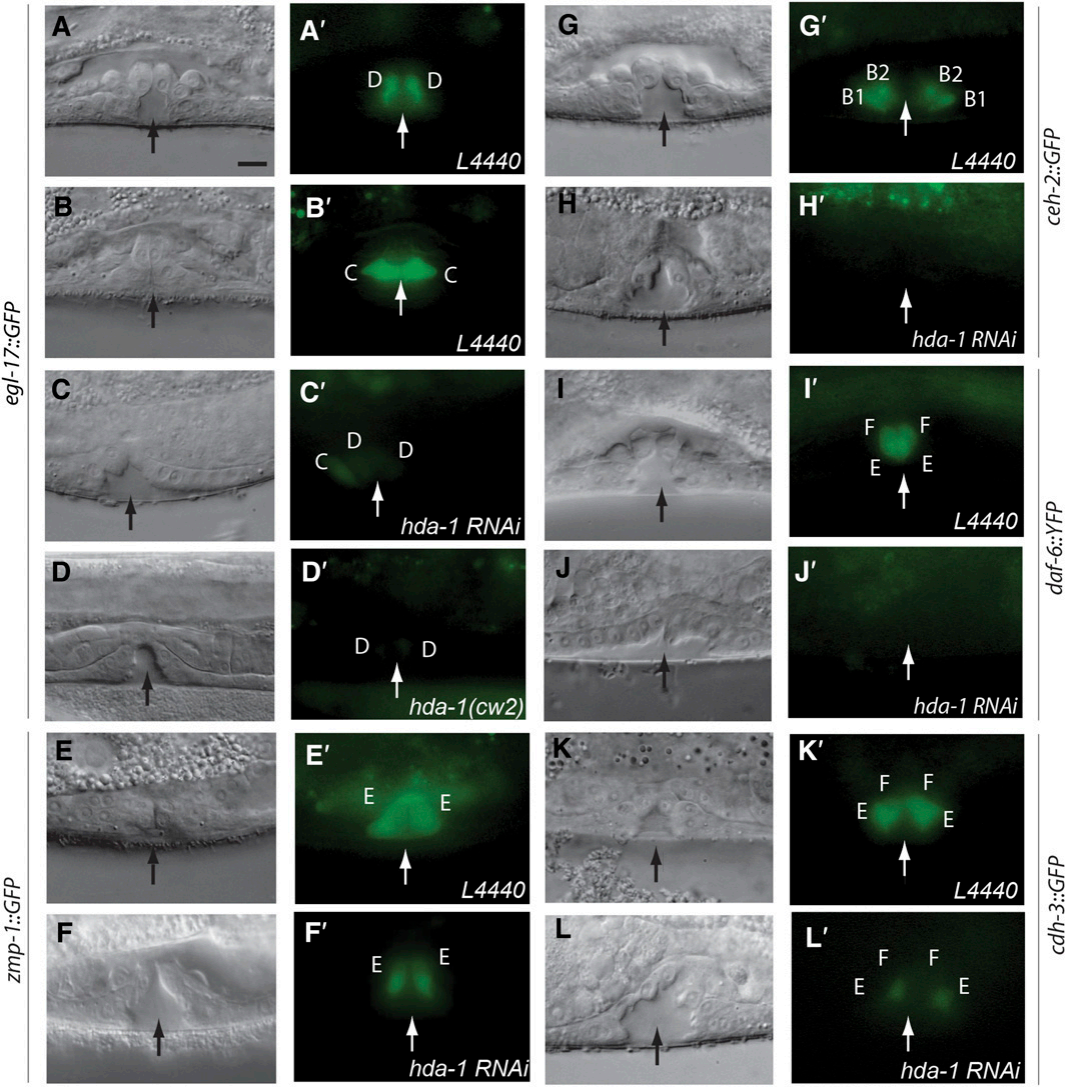
Vulval cell fate specification defects in *hda-1 (RNAi)* animals. (A−L) Nomarski images of L4 stage vulval cells. (A′−L′) Corresponding GFP fluorescence photomicrographs. (A−D, A′-D′) *egl-17*::*gfp (ayIs4)*; (E−F, E′−F′) *zmp-1*::*gfp (syIs49)*; (G, H, G′, H’) *ceh-2*::*gfp (syIs54)*; (I, J, I′, J′) *daf-6*::*yfp (bhEx53)* and (K−L, K′−L′) *cdh-3*::*gfp (syIs50)*. The expression patterns of all markers are affected in *hda-1* animals. Arrows mark the center of vulval invagination. B1, B2, C, D, E, and F refer to the presumptive vulval cell fates vulB1, vulB2, vulC, vulD, vulE, and vulF, respectively. Scale bar is 10 μm.

The abnormal vulval morphology and Pvl phenotype in the *hda-1* animals, together with defective *ajm-1*::*gfp* toroids, led us to further characterize the role of *hda-1* in vulval development. For this, we used five vulval cell type-specific GFP-based markers, *zmp-1*::*gfp* (zinc metalloproteinase), *egl-17*::*gfp* (fibroblast growth factor family), *ceh-2*::*gfp* (homeobox family), *daf-6*::*yfp* (patched family), and *cdh-3*::*gfp* (Fat cadherin family), which are expressed in subsets of differentiating vulval cells (Inoue *et al.* 2002; Perens and Shaham 2005). *egl-17*::*gfp* expression was first observed in mid-L3 animals in P6.p granddaughters, and later, in mid-L4 animals in the presumptive vulC and vulD cells (Figure 2A, A′, and B, B′). *ceh-2*::*gfp* and *daf-6*::*yfp* showed a more restricted pattern of expression. Although *ceh-2*::*gfp* was observed in the presumptive vulB1 and vulB2 cells (2° lineage) (Figure 2, G and G′), *daf-6*::*yfp* was observed in the presumptive vulE and vulF cells (1° lineage cells; Figure 2, I and I′). The remaining two markers, *zmp-1*::*gfp* and *cdh-3*::*gfp*, showed GFP fluorescence in subsets of both 1° and 2° lineage cells. *cdh-3*::*gfp* was expressed in presumptive vulE, vulF cells (Figure 2, K and K′), vulC and vulD (not shown) whereas *zmp-1*::*gfp* was observed in vulE (Figure 2, E and E′), vulA and vulD cells (not shown).

The analysis of the aforementioned markers in *hda-1* animals revealed defects in cell type-specific gene expression (Table 2). Specifically, *egl-17*::*gfp* fluorescence was weak and often absent in both the *hda-1(cw2)* and *hda-1(RNAi)* animals (Figure 2, C, C′ and D, D′). The *zmp-1*::*gfp* level was significantly reduced in presumptive vulE cells (Figure 2, F and F′). The levels of *ceh-2*::*gfp* and *daf-6*::*yfp* were frequently below the detectable limit (Figure 2, H, H′ and J, J′), whereas *cdh-3*::*gfp* was often reduced in the mutants (see vulF in Figure 2, L and L′) or missing (not shown). Changes in marker gene expression revealed that the specification of all vulval progeny was affected. We did not observe any case of VPC fate transformation, *i.e.*, 1° to 2° or vice-versa. These results, together with the abnormal vulval toroids and defects in invagination in *hda-1* mutant animals (Figure 1I), demonstrated that *hda-1* is necessary for the differentiation as well as correct division patterns of both 1° and 2° lineage cells.

**Table 2:** Vulval cell fate specification defects in *hda-1* RNAi animals

Cell Fate Marker	RNAi	Vulval Cell Type						
		A	B1/2	C	D	E	F	n
*zmp-1*::*gfp*	*L4440*	100%		100%	100%	100%		50
	*hda-1*	ND		83.3%	100%	62.5%		24
*ceh-2*::*gfp*	*L4440*		100%					50
	*hda-1*		81%					27
*egl-17*::*gfp*	*L4440*			100%	100%			50
	*hda-1*			60%	60%			30
*cdh-3*::*gfp*	*L4440*			100%	100%	100%	100%	50
	*hda-1*			66.6%	100%	66.6%	40%	15
*daf-6*::*yfp*	*L4440*					100%	100%	50
	*hda-1*					76.6%	83.3%	30

Percentage of vulval cells having GFP fluorescence are shown. A, C, D, E, and F refer to the presumptive vulval cell fates vulA, vulC, vulD, vulE, and vulF, respectively. vulB1, and vulB2 are listed together as B1/2. L4440 refers to control RNAi animals. RNAi, RNA interference; n, number of animals examined; ND, not done.

**Figure 3:**
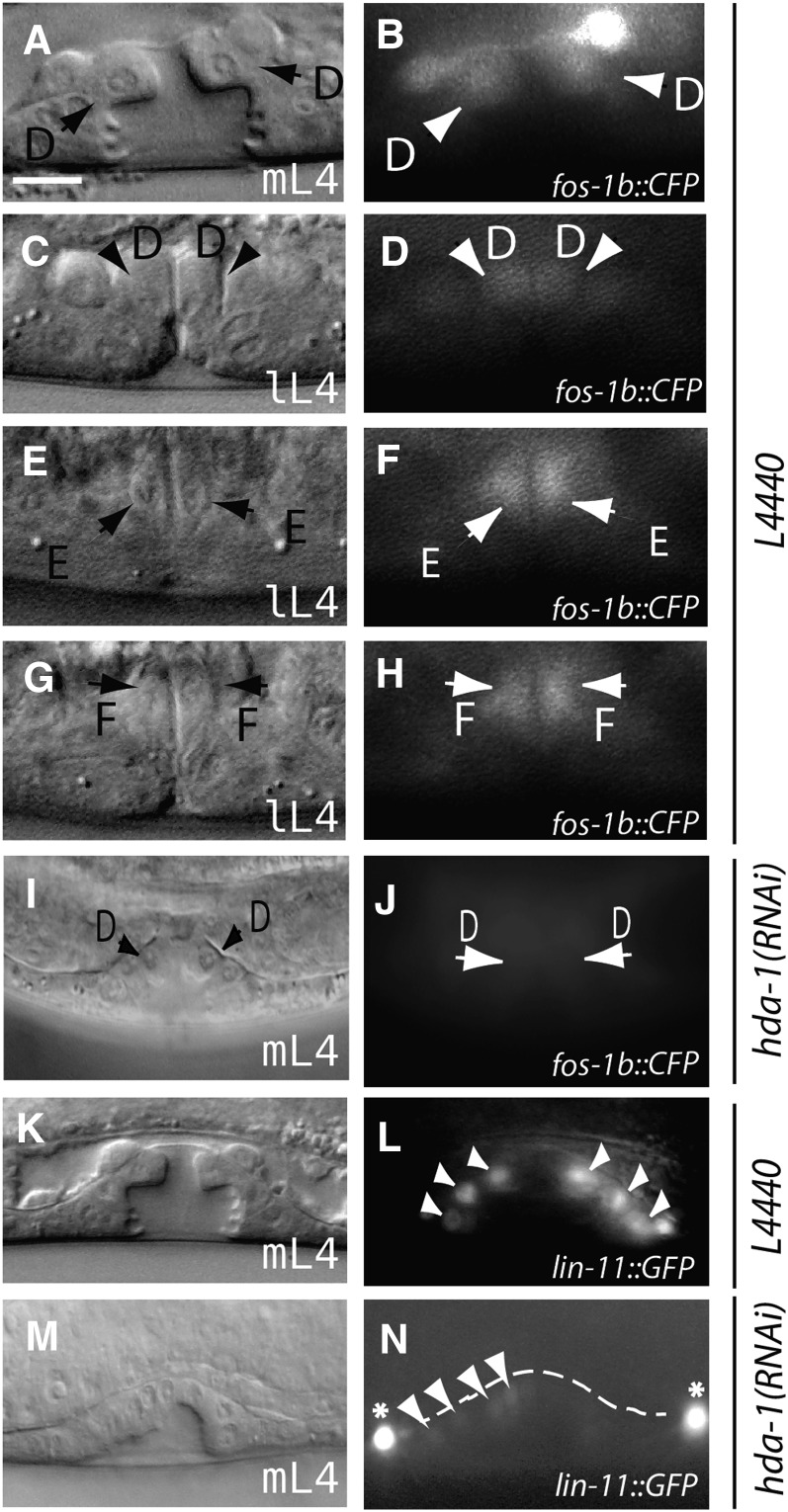
*lin-11* and *fos-1* expression is altered in *hda-1* mutants. DIC and corresponding fluorescent images of animals expressing a translational *fos-1*::*cfp* reporter. (A and B) Control L4440 RNAi-treated mid-L4 animal showing *fos-1* expression in presumptive vulD cells. (C−H) mid/late-L4 stage animals showing *fos-1* expression in presumptive vulD, vulE and vulF cells. (I, J) *hda-1* knockdown causes reduction in *fos-1*::*cfp* expression. Diffuse CFP fluorescence is observed in the region overlapping with presumptive vulD cells. *lin-11* expression is detected in vulval cells in control RNAi-treated animals (K, L) but is absent in *hda-1-RNAi* treated animals (M, N). Some of the GFP fluorescing cells are marked by arrowheads and arrows (D, E and F refer to vulD, vulE and vulF, respectively). mL4: mid-L4, lL4: late-L4. Asterisk in panel N points to VC neuronal cells. Scale bar is 10 μm.

We also examined the expression of two transcription factors, *lin-11* and *fos-1*, in *hda-1(RNAi)* animals. Both these genes are involved in vulval morphogenesis (Gupta *et al.* 2003; Seydoux *et al.* 1993). *lin-11* is expressed in all vulval progeny while cells are differentiating and undergoing morphogenetic changes (Gupta *et al.* 2003). The *fos-1* locus encodes three transcripts that have some functional differences. *fos-1a (syIs123 fos-1a*::*yfp)* is almost exclusively present in the AC and is the target of *hda-1* during AC invasion (Matus *et al.* 2010). *fos-1b(syIs137fos-1b*::*cfp)* is observed at a low level in several uterine cells, including the AC (Sherwood *et al.* 2005), and it does not appear to play a role in AC invasion. Another *fos-1* transcript, *fos-1c*, is expressed in uterine π lineage cells and involved in utse formation (Oommen and Newman 2007). We examined *syIs123* and *syIs137* transgenic animals and found that although *fos-1a*::*yfp* was undetectable in vulval cells during the L3 and L4 stages (data not shown), *fos-1b*::*cfp* was expressed in a subset of vulval progeny. During the mid-L4 stage, CFP fluorescence was brighter in presumptive vulD cells compared with vulE and vulF cells (Figure 3, A and B). This pattern was dynamic, such that by late-L4 stage, the presumptive vulE and vulF cells were much brighter compared with the presumptive vulD cells (Figure 3, C−H).

We found that *lin-11*::*gfp (syIs80)* expression was significantly reduced in *hda-1(RNAi)* animals (74% faint and 26% animals with no GFP fluorescence, n = 53%; Figure 3, K−N). Expression was uniformly lower, consistent with *hda-1* expression requirements in all vulval progeny. Similar to *lin-11*, *fos-1b*::*cfp* fluorescence was also reduced. In mid-L4 animals, the presumptive vulE and vulF cells showed almost no fluorescence, whereas presumptive vulD cells were faintly visible (78% animals defective, n = 16, compared with none in control, n = 25) (Figure 3, I and J). The pattern was similar in late-L4 animals (data not shown). These results demonstrate the importance of *hda-1* in regulating *lin-11* and *fos-1b* in vulval cells.

### *hda-1* is expressed in vulval and gonadal lineage cells

To further characterize the role of *hda-1* in reproductive system development, we examined its expression profile by using the *gfp* reporter transgenic strains *sEx13706* and *bhEx72*. The *sEx13706* strain was generated earlier as part of a systematic gene expression-profiling project (Hunt-Newbury *et al.* 2007). Expression of *gfp* in *sEx13706* animals is directed by a 2.8-kb *hda-1* regulatory region that includes the open reading frames and potential *cis*-regulatory elements (enhancers) of two other *hda-1* upstream genes (*ril-1* and *C53A5.2*; Figure S2). The other *hda-1*::*gfp* transgenic strain (*bhEx72*), which was generated by us, contains a much smaller 5′ upstream region of *hda-1* (approximately 1.0 kb, pGLC44) and excludes the two genes mentioned above (Figure S2A, also see the *Materials and Methods* section). The analysis of GFP fluorescence in *sEx13706* and *bhEx72* animals revealed a similar pattern, although the fluorescence in *sEx13706* was much brighter. We found that *hda-1* is broadly expressed throughout development (Figure S2, B−O). The earliest expression was detected in gastrulating embryos. The larvae exhibited GFP expression in several neuronal and epidermal cells, primarily in the anterior ganglion and ventral hypodermal regions. Expression persisted in many cells in later larval and adult stages (data not shown).

**Figure 4:**
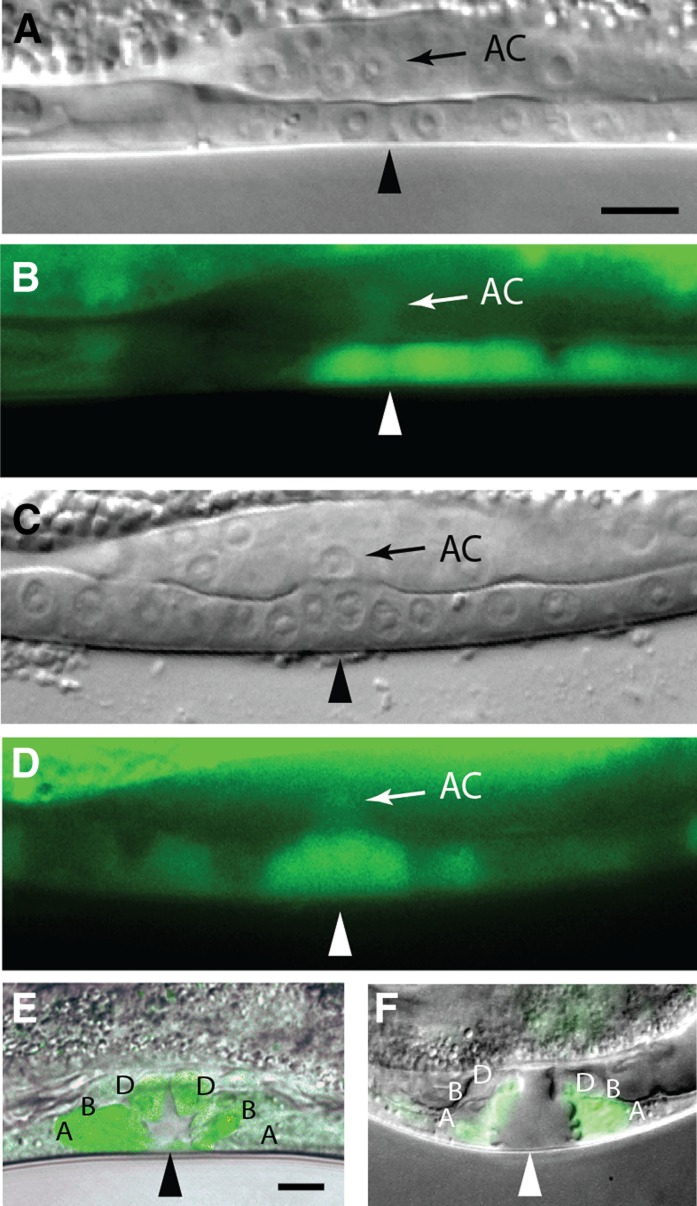
*hda-1* expression in the vulva and AC. (A−E) *sEx13706* and (F) *bhEx68*. (A, B) Pn.px cells. (C, D) Pn.pxx cells. (E, F) Pn.pxxx cells. Triangles mark the center of vulval invagination. The presumptive vulval cell types A (vulA), B (vulB1 or vulB2), and D (vulD) are shown. The AC is shown with arrows. In (B), P5.p is in the process of dividing and has reduced level of GFP fluorescence. Scale bar is 10 μm.

In the vulva, *hda-1*::*gfp* expression was first detected in the progeny of P(5-7).p in mid-L3 animals (Figure 4, B and D). At this stage, GFP fluorescence was absent in other VPC lineages (P3.p, P4.p and P8.p; data not shown). By the L4 stage, almost all vulval cell types were observed fluorescing, with presumptive vulA, vulB1, vulB2, and vulD cells being the brightest (Figure 4E). GFP fluorescence in vulval cells was mostly absent beyond the late-L4 stage, suggesting that *hda-1* may not be needed in vulval cells at later stages of development. The broad expression of *hda-1* is consistent with the involvement of the gene in multiple developmental processes. This multifaceted role for *hda-1* in *C. elegans* appears to be conserved in *C. briggsae* because *Cbr-hda-1*::*gfp* is expressed in a similar manner (Figure 4F and data not shown).

We also observed *hda-1*::*gfp* expression in the AC in L3 animals (Figure 4, B and D) that persisted until the early L4-stage (data not shown). No expression was observed in π cells or their progeny at any developmental stage. Considering that AC movement and the vulval-uterine connection are abnormal in *hda-1* mutants (Figure 1, B−E), a simple model could be that *hda-1* acts in the AC to control π cell fates and utse formation. The experiments described in the sections to follow support this model.

### *hda-1* mutants exhibit defects in the specification of uterine π lineage cells

In addition to the vulval defect, *hda-1* mutants also lack a functional vulval−uterine connection, as the thin utse membrane-like structure could not be clearly identified in these animals (see Figure 1). In wild-type L3 stage animals, three VU cells divide to produce 12 granddaughters, six of which are induced by the AC to adopt π fates (located in two different focal planes, three on each side). By the early L4 stage, π cells produce 12 daughters, eight of which fuse with each other and the AC to form the utse (Newman *et al.* 1996). This process is controlled by a number of genes, including the transcription factors *egl-13* and *lin-11*. These two genes play important roles in π cell differentiation and utse formation (Hanna-Rose and Han 1999; Newman *et al.* 1999).

**Figure 5:**
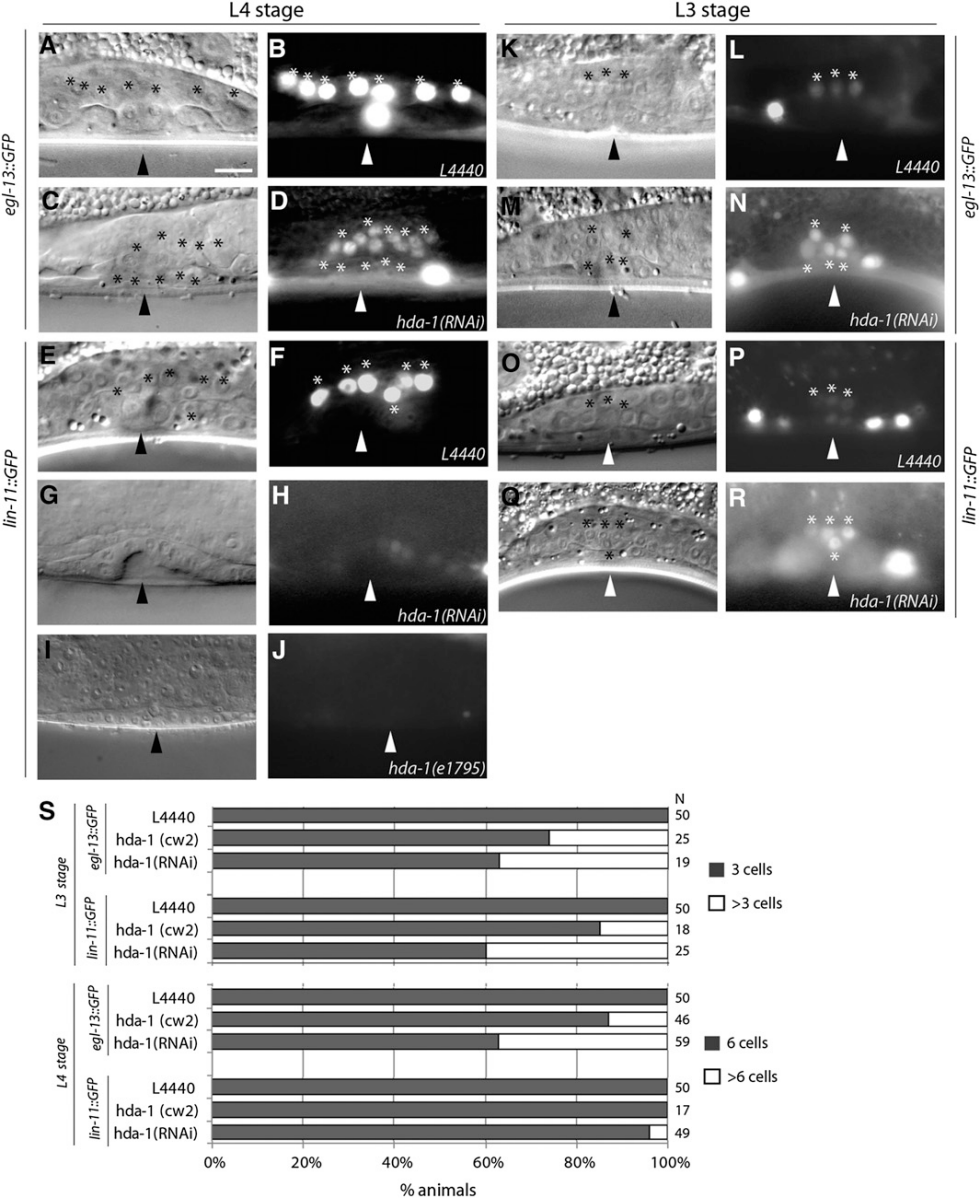
π fate specification defects in *hda-1* animals. Animal stages and transgenes are shown on the lateral side of the images and genotypes on the bottom of each image. Arrowheads mark the center of vulval invagination. π cells and their progeny are indicated by asterisks. (A, B) In a wild-type *egl-13*::*gfp* L4 animal, 7 *gfp*-expressing cells (6 π progeny and the AC) are visible. (E, F) A *lin-11*::*gfp* animal of similar age shows 6 π progeny in this focal plane. (C, D) *hda-1* RNAi causes an increase in π cells. An *egl-13*::*gfp* animal showing 10 π progeny following *hda-1* knockdown. (G, H) Similar knockdown in a *lin-11*::*gfp* strain results in significant reduction in GFP fluorescence in vulval cells. The π progeny in this animal are too faint to see. (I, J) The *e1795* allele of *hda-1* causes greater reduction in *lin-11:gfp* expression. In this animal, no fluorescence is visible in the vulva or uterine cells. π cells in *egl-13*::*gfp* (K, L) and *lin-11*::*gfp* (O, P) animals. (M, N and Q, R) An increased number of π cells are observed in *egl-13*::*gfp* and *lin-11*::*gfp* animals following *hda-1* knockdown. (S) Quantification of *egl-13*::*gfp and lin-11*::*gfp* expressing cells in late-L3 and early/mid-L4 stage animals. The percentage of animals is shown on the *x*-axis, whereas genotypes are indicated on the *y*-axis. N = number of animals examined; Scale bar (A−R) is 10 μm.

To characterize the utse defect in *hda-1* animals, we examined *egl-13* and *lin-11* expression in π lineage cells using *GFP* reporter-expressing transgenic strains (*egl-13*::*gfp kuIs29* and *lin-11*::*gfp syIs80)*. In wild-type animals, both genes are expressed in π cells and their progeny (Figure 5, A, B, E, F, K, L, O, and P) (Gupta and Sternberg 2002; Hanna-Rose and Han 1999). We found that *hda-1(RNAi)* and *hda-1(cw2)* animals have abnormal patterns of *egl-13*::*gfp* and *lin-11*::*gfp* expression. Specifically, there were more GFP-fluorescing π-like cells (as many as seven) in the mutants (Figure 5, N, R, and S), suggesting that the VU granddaughters failed to limit the expression of *egl-13* and *lin-11* in *hda-1* mutants. Similar to π cells, the number of π progeny also was greater (up to 13) (Figure 5, D and S), although in the case of *lin-11*::*gfp*, the overall level of GFP fluorescence was considerably reduced (RNAi-treated: 74% faint and 26% absent, n = 53 animals; *e1795*: 100% absent, n = 21) (Figure 5, G−J). The π progeny failed to migrate as they normally do in wild-type animals. As *egl-13* controls π cell divisions and the number of π progeny (Hanna-Rose and Han 1999), it is conceivable that extra π progeny in *hda-1* animals arise in part from a reduction in *egl-13* expression. In summary, these results suggest that although more π-like cells are formed in *hda-1* mutants, the cells fail to differentiate correctly, resulting in the lack of a functional vulval-uterine connection.

**Figure 6:**
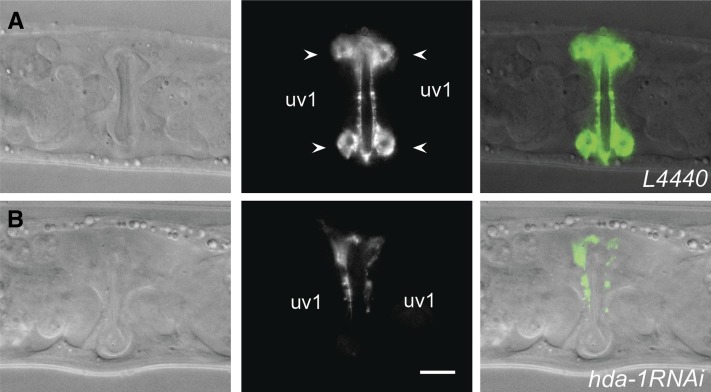
uv1 differentiation defect in *hda-1(RNAi)* animals. Nomarski (left), fluorescence (middle), and overlapping (right) images of late-L4 stage animals expressing *ida-1*::*gfp* in the uv1 cells (arrow) of the ventral uterus. (A) Four uv1 cells are observed in L4440 control RNAi-treated animals. (B) No uv1 cells are visible in this *hda-1*(RNAi) animal. Scale bar is 20 μm.

We also examined uv1 cell fate in *hda-1* mutants. uv1 cells are specified from among the progeny of π cells during the L3 lethargus stage (Newman *et al.* 1996). Examination of the uv1-specific marker *ida-1*::*gfp* (Zahn *et al.* 2001) revealed that unlike wild-type animals in which four uv1 cells were visible (Figure 6A), 96% (n = 160) *hda-1* mutants showed no such expression, suggesting there is a defect in uv1 differentiation (Figure 6B). Taken together, these results demonstrated that *hda-1* plays an important role in π lineage specification, leading to the formation of utse and uv1 cells.

### *hda-1* mutants show defects in AC fate and fail to regulate *lag-2* expression

**Figure 7:**
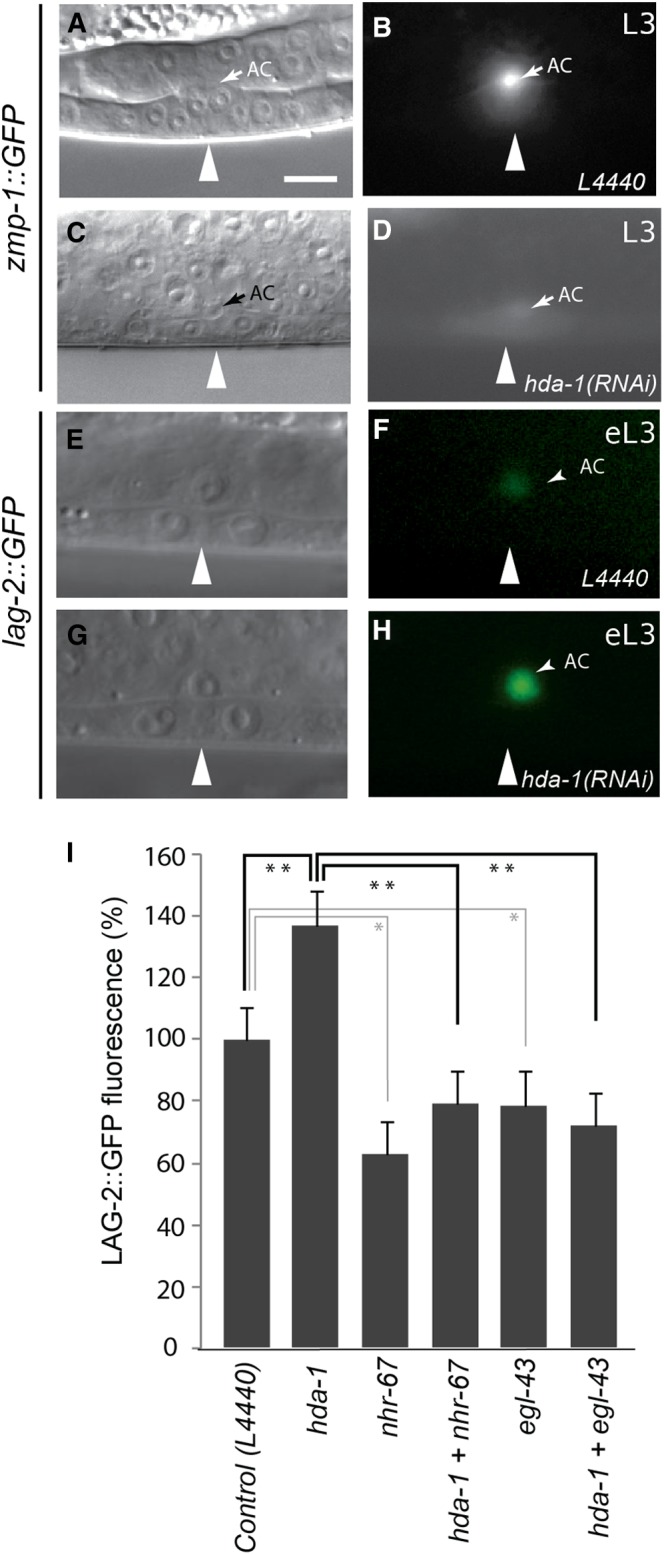
Effect of *hda-1* RNAi knockdown on the AC. (A, B) *zmp-1*::*gfp* expression in the AC of a wild-type animal. (C, D) *zmp-1* expression is strongly diminished in *hda-1(RNAi)* animal. (E, F) Wild-type *lag-2*::*gfp* (arEx1352) expression in the AC. (G, H) GFP fluorescence in AC is brighter in *hda-1(RNAi)* animal. Arrowheads mark the center of vulval invagination. Scale bar is 10 μm. (I) Quantification of *lag-2*::*gfp* fluorescence intensity in the AC. The *hda-1*(RNAi) animals show a significant increase in GFP fluorescence compared with controls. In contrast, *nhr-67*(RNAi) and *egl-43*(RNAi) animals show reduced GFP fluorescence in the AC. The increase in *lag-2*::*gfp* fluorescence in *hda-1(RNAi)* animals was suppressed by *nhr-67(RNAi)* and *egl-43(RNAi)*. 20 or more animals were examined in each case. eL3, early-L3. The *P* values for pairs are indicated by stars (**P < 0.01, *P < 0.05).

The expression of *hda-1* in the AC and its requirement for AC migration suggested to us that the utse defect in *hda-1* animals might be caused by a failure in AC differentiation. Earlier, *hda-1* was shown to be required in the AC for cell invasion and expression of *lin-3*::*gfp* (EGF ligand) (Matus *et al.* 2010); however, the role of *hda-1* in the AC-mediated utse differentiation process was not investigated. Therefore, we first examined AC fate using a *zmp-1*::*gfp (syIs49)* reporter strain. *zmp-1* is expressed in the AC starting at L3 and is involved in AC function (Rimann and Hajnal 2007; Sherwood *et al.* 2005). RNAi-mediated knockdown of *hda-1* caused a significant reduction in GFP fluorescence in the *zmp-1*::*gfp* animals (Figure 7, A−D, 100% bright in control, n = 35; 64% reduced and 0% absent in *hda-1(RNAi)*, n = 58; 25% reduced and 70% absent in *e1795*, n= 20), suggesting that the AC was defective in *hda-1* animals.

Next, we examined AC-mediated signaling by investigating the expression of *lag-2*. LAG-2 is a DSL ligand expressed in the AC, and it mediates *lin-12/Notch* signaling in the presumptive π cells (Newman *et al.* 2000). The *hda-1(e1795*) animals were previously shown to have ectopic *lag-2*::*gfp* fluorescence in certain unidentified cells beneath the cuticle, suggesting that *hda-1* normally represses *lag-2* in these cells (Dufourcq *et al.* 2002). We reasoned that an increase in π cell numbers in the *hda-1* mutants could be caused by the over expression of *lag-2* in the AC, leading to the inappropriate activation of *lin-12/Notch* signaling in VU granddaughters. This is in line with previous findings that showed an increase in π cells in *lin-12* gain-of-function (*gf*) mutants in which *lin-12* receptor activity is elevated and operates in a ligand-independent manner (Newman *et al.* 2000). Therefore, we quantified GFP fluorescence in the AC in *lag-2*::*gfp* animals at the time of π cell induction. As expected, *hda-1*(RNAi) animals exhibited a much higher level of GFP fluorescence in the AC compared with controls (average increase of 37% ± 9%, n = 30) (Figure 7, E−I).

### *nhr-67* and *egl-43* act downstream of *hda-1* to promote *lag*-2 expression in the AC and specify π cells

**Figure 8:**
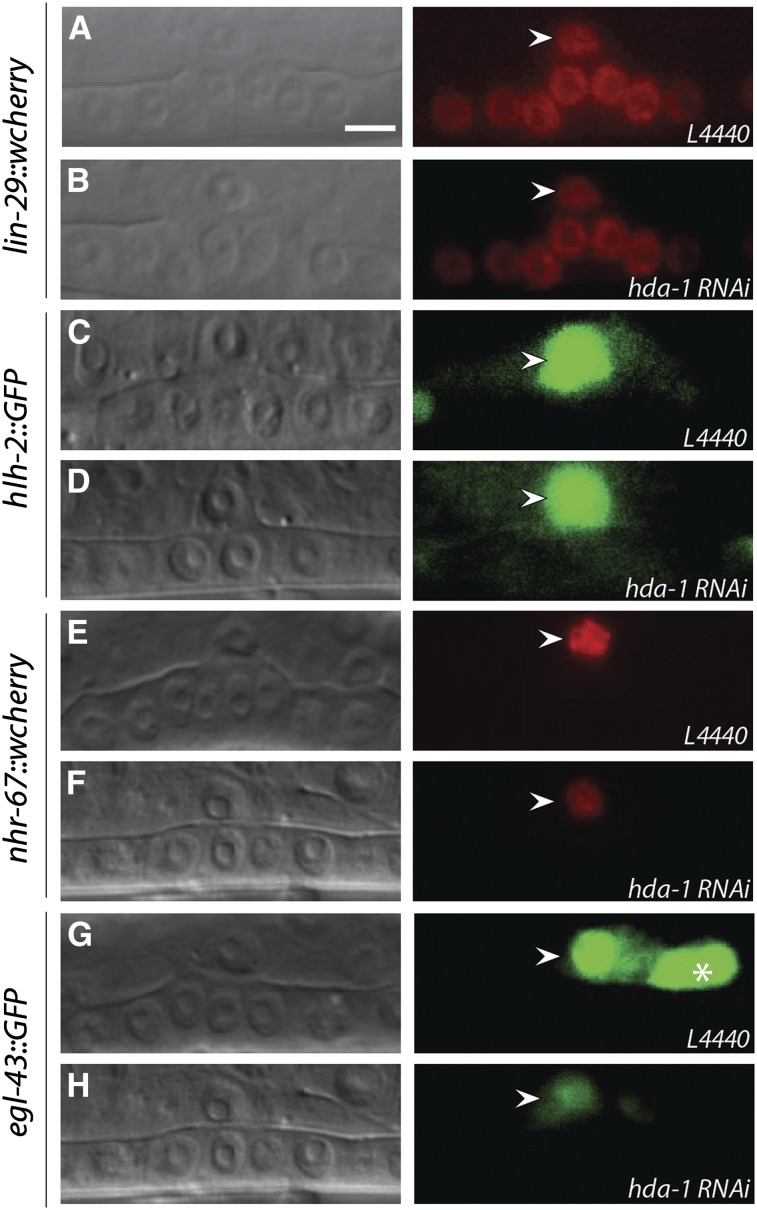
Effect of *hda-1(RNAi)* on AC expression of transcription factors. Transgenic animals with fluorescent reporters for *lin-29* (A, B), *hlh-2* (C, D), *nhr-67* (E, F), and *egl-43* (G, H) were treated with either control L4440 or *hda-1* RNAi. Nomarski images are on the left, and the corresponding fluorescence images are on the right. GFP fluorescence is unaltered in *lin-29*::*wcherry* and *hlh-2*::*gfp* animals. However, *nhr-67*::*wcherry* and *egl-43*::*gfp* fluorescence in the AC is reduced. *lin-29*::*wcherry* expression is also observed in vulval lineage cells. Arrowheads mark the AC and the star in G points to a VU cell. 20 or more animals were examined in each case. Scale bar is 5 μm.

The up-regulation of *lag-2*::*gfp* in the AC in *hda-1* mutant animals prompted us to search for genes involved in *hda-1*-mediated *lag-2* repression. To pursue this goal, we investigated the roles of four transcription factors: *hlh-2* (bHLH family, *E/daughterless* homolog), *lin-29* (C2H2 Zinc finger family), *nhr-67*, and *egl-43*. All of these genes are expressed in the AC, and except for *egl-43*, have been shown to positively regulate *lag-2* expression (Karp and Greenwald 2003; Newman *et al.* 2000; Verghese *et al.* 2011). We found that the expression of *hlh-2*::*gfp* and *lin-29*::*wcherry* in the AC was unaltered in *hda-1*(RNAi) animals, but *nhr-67*::*wcherry* and *egl-43*::*gfp* fluorescence was reduced (Figure 8). These results suggest that *hda-1* positively regulates the expression of *nhr-67* and *egl-43* in the AC. The other two genes, *hlh-2* and *lin-29*, function in an *hda-1*−independent manner.

**Figure 9:**
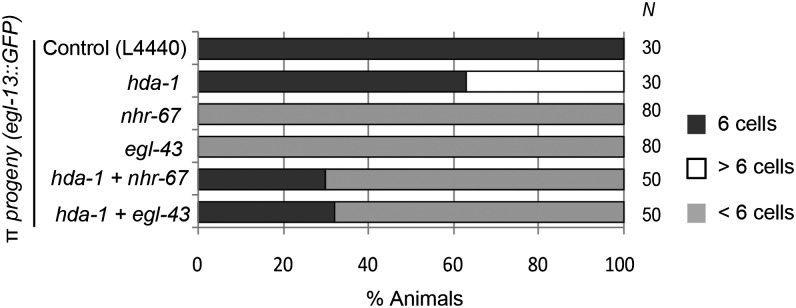
π cell fate defects following knockdowns of *hda-1*, *nhr-67*, and *egl-43*. π cells were counted in the early to mid-L4 stages in single and double RNAi-treated animals. The percentage of animals is plotted. *nhr-67* and *egl-43* suppress the extra π cell phenotype caused by the reduction of *hda-1* function. The number of animals in each case (N) is shown.

**Figure 10:**
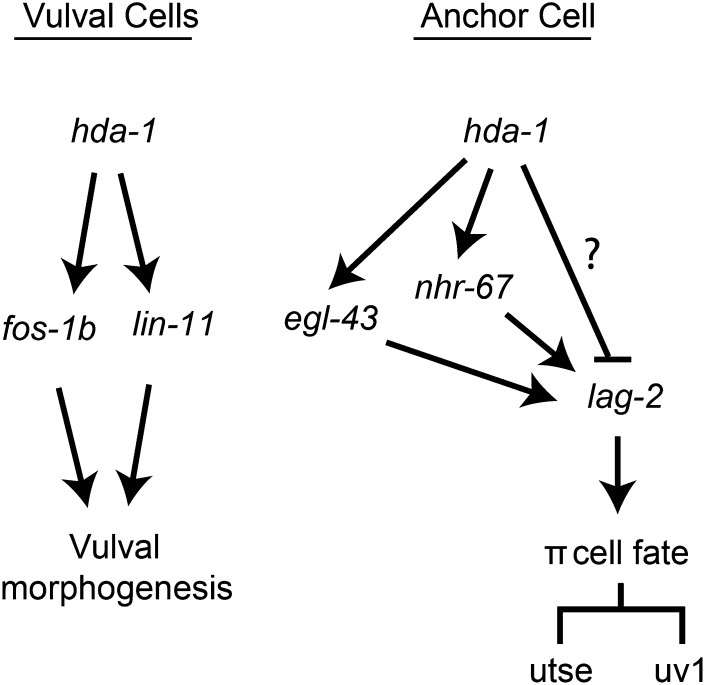
A model for *hda-1* function in *C. elegans* reproductive system development. The model has two parts. In the first part, *hda-1* is expressed in vulval cells and regulates *fos-1b* and *lin-11* to control vulval morphogenesis. In the second part, *hda-1* acts in the AC to specify π cell fates to give rise to utse and uv1 cells. This process is mediated by *lag-2*, which is both positively and negatively regulated by *hda-1*. In the case of positive regulation, *hda-1* interacts with *nhr-67* and *egl-43*. The factor(s) mediating negative regulation of *lag*-2 (indicated by the question mark) are unknown.

Next, we investigated whether *hda-1* regulates the expression of *nhr-67* and *egl-43* in the AC to specify π cell fates. One possibility is that these two genes act downstream of *hda-1* to repress *lag-2* transcription. Interestingly, RNAi-mediated knockdown of *nhr-67* or *egl-43*, either alone or in combination with *hda-1*, caused a significant reduction in *lag-2*::*gfp* fluorescence in the AC (Figure 7I). The *lag-2*::*gfp* de-repression phenotype of *hda-1(RNAi)* was fully suppressed by *nhr-67(RNAi)* and *egl-43(RNAi)*, suggesting that both transcription factors are necessary for *hda-1*-mediated *lag-2* regulation. As expected, the mutant animals also had fewer π cells, as revealed by *egl-13*::*gfp* expression (Figure 9). Taken together, these findings allowed us to conclude that *nhr-67* and *egl-43* act downstream of *hda-1* to promote *lag-2* expression and π cell fate specification. However, they do not rule out the possibility that *hda-1* and *nhr-67* act independently in parallel to regulate *lag-2* expression in the AC. Furthermore, these results suggest that other unidentified factors might also be involved in mediating *hda-1* function in this process (Figure 10).

## Discussion

HDAC1 family members are present in diverse animal phyla and control a wide range of developmental processes. In *C. elegans*, HDA-1 has been shown to function as a transcriptional repressor and is involved in embryogenesis, gonadogenesis, germline formation, and vulval cell proliferation (Calvo *et al.* 2001; Dufourcq *et al.* 2002; Solari and Ahringer 2000; Zinovyeva *et al.* 2006). In this study, we report new, previously unidentified roles for *hda-1* in the specification of the vulva and uterine π cell fates and describe the genetic basis of its function in these two lineages.

### *hda-1* controls vulval morphogenesis

Previously, *hda-1* was shown to be required for vulval invagination, possibly by controlling the division axes of certain vulval cells (Dufourcq *et al.* 2002). We used five GFP-based cell fate markers to characterize the vulva phenotype in mutant animals and found that the cells in *hda-1* animals failed to acquire correct identities. We also used a cell junction marker, *ajm-1*::*gfp*, to examine vulval toroids and found wider and sometimes missing rings, which is consistent with altered cell fates in *hda-1* animals. In addition to cell fate specification studies, we also examined *hda-1*::*gfp* expression during development. GFP fluorescence was first detected in P(5−7).p daughters, and the expression continued in their progeny in the L3 and L4 stages, when cells acquire a specific fate (vulA to F) and undergo morphogenetic changes. Together, these results demonstrate the importance of *hda-1* in vulval morphogenesis.

To identify *hda-1* targets, we investigated the roles of two important transcription factors, *lin-11* (LIM-HOX family) and *fos-1b* (*fos* proto-oncogene family). Mutations in these two genes cause defects in the differentiation and invagination of vulval progeny (Ferguson *et al.* 1987; Gupta *et al.* 2003; Marri and Gupta 2009; Seydoux *et al.* 1993). Our finding that *hda-1* is required for the expression of *lin-11*::*gfp* and *fos-1b*::*cfp* in vulval cells provides evidence that *hda-1* act upstream of both genes in vulval morphogenesis.

### *hda-1* is necessary for utse differentiation

We uncovered a new role for *hda-1* in the formation of the vulval-uterine connection. Unlike in the wild-type animals, a thin utse membrane above the vulva cannot be observed in *hda-1* animals. Our results showed that this defect is caused by the misspecification of π cell fates, as assessed by the expression of the transcription factors *lin-11* and *egl-13*. The *hda-1* mutants showed an increased number of π cells, suggesting that *hda-1* normally limits the π fate of VU granddaughters. This defect was accompanied by the lack of uv1, as determined by the analysis of *ida-1*::*gfp* marker expression. Because VU precursors divide to give rise to the π cells that ultimately form the utse and uv1, these results demonstrate that *hda-1* plays an important role in VU lineage specification.

The π cell phenotype in *hda-1* animals is caused by defects in AC differentiation. We found that *hda-1* is expressed in the AC at the time of π cell fate specification. Additionally, *zmp-1*::*gfp* expression was not observed in the AC of *hda-1* mutants. These results, in combination with those involving the role of *hda-1* in AC invasion (Matus *et al.* 2010), demonstrate a broad requirement for *hda-1* in AC-mediated processes.

Genetic studies have shown that AC-mediated LIN-12/Notch signaling is necessary for the specification of π cell fate. The AC produces the DSL ligand *lag-2*, which activates the *lin-12* pathway in VU cells. Therefore, alterations in *lag-2* expression are likely to impact *lin-12* signaling and π cell fate specification process. To address the role of *hda-1* in utse formation, we examined the *lag-2*::*gfp* pattern in the AC and found it to be de-repressed in *hda-1(RNAi*) animals. Thus, *hda-1* appears to limit the level of *lag-2* transcription in the AC, thereby preventing inappropriate activation of LIN-12/Notch signaling in VU cells. We have found evidence for both positive and negative control mechanisms in *hda-1*−mediated regulation of *lag-2*. Although the genes that negatively regulate *lag-2* expression are currently unknown, the positive regulation of *lag-2* involves two important transcription factors: *egl-43* and *nhr-67* (Figure 10). The roles of *egl-43* and *nhr-67* have been studied previously in different developmental contexts. In the reproductive system, *egl-43* regulates *nhr-67* expression in the AC and *nhr-67* in turn regulates *lag-2*-mediated AC and utse fate specification (Rimann and Hajnal 2007; Verghese *et al.* 2011). However, their relationship with *hda-1* was unknown. Our study provides the first genetic evidence of an interaction between *hda-1*, *nhr-67*, and *egl-43* in AC-mediated π cell fate specification processes. More work is needed to understand the precise nature of the interactions between these three genes.

In summary, we have demonstrated the crucial role of *hda-1* in regulating LIN-12/Notch signaling in π fate specification. Antagonistic interactions between HDAC1 and the Notch pathway have been previously observed in various developmental contexts, such as neurogenesis and smooth muscle differentiation (Cunliffe 2004; Tang *et al.* 2012; Yamaguchi *et al.* 2005). Although the molecular basis of the HDAC1−Notch interaction remains unclear, HDAC1 co-repressor complexes (*e.g.*, NURD) may play a role in some cases (Cunliffe 2008; Hayakawa and Nakayama 2011). Further analysis of the role of *hda-1* in π fate specification processes could help clarify the mechanism of interaction between *hda-1* and the LIN-12/Notch pathway.

### HDAC1 and NURD complex genes in reproductive system development in *C*. *elegans*

Studies of HDAC1 have shown that it is part of the NURD protein complex that controls gene transcription by altering chromatin structure (Denslow and Wade 2007). Other NURD complex components include Mi2 ATPase, retinoblastoma-associated factors RbAp46/48, metastasis tumor associated factor, and the accessory protein p66. The *C. elegans* genome contains corresponding family members of these genes, all of which play important roles in the formation of the vulva and in other developmental processes (Dufourcq *et al.* 2002; Herman *et al.* 1999; Poulin *et al.* 2005; Unhavaithaya *et al.* 2002; von Zelewsky *et al.* 2000; Zhao *et al.* 2005). Because most *C. elegans* NURD genes are members of the SynMuv family, which interacts with Ras pathway components, their function has been primarily studied in the context of Ras-mediated vulval cell proliferation (Fay and Yochem 2007). Whether these genes have additional roles in the vulva and uterus has yet to be fully explored. von Zelewsky *et al.* (2000) previously showed that mutations in the Mi2 genes *let-418* and *chd-3* affect cell division and the invagination of vulval cells. Together with our work on *hda-1*, these results lend support to the conclusion that the NURD complex components play important roles in the morphogenesis of the vulva and vulva-uterine connection. In the future, characterization of *hda-1* interactions with other NURD components should reveal whether *hda-1* acts as part of the chromatin complex or through some other mechanism in reproductive system morphogenesis. The results will ultimately contribute to a better understanding of HDAC1-mediated gene regulation events in *C. elegans* and other eukaryotes.

## Supplementary Material

Supporting Information

## References

[bib1] Brenner S., 1974 The genetics of *Caenorhabditis elegans*. Genetics 77: 71–94 4366476 10.1093/genetics/77.1.71 PMC1213120

[bib2] Calvo D. Victor M. Gay F. Sui G. Luke M. P., 2001 A POP-1 repressor complex restricts inappropriate cell type-specific gene transcription during Caenorhabditis elegans embryogenesis. EMBO J. 20: 7197–7208 11742996 10.1093/emboj/20.24.7197 PMC125335

[bib3] Cui, M., and M. Han, 2007 Roles of chromatin factors in *C. elegans* development. *WormBook*, ed. The *C. elegans* Research Community WormBook, /10.1895/wormbook.1.139.1. Available at http://www.wormbook.org 10.1895/wormbook.1.139.1 PMC4781364 18050494

[bib4] Cui M. Chen J. Myers T. R. Hwang B. J. Sternberg P. W., 2006 SynMuv genes redundantly inhibit lin-3/EGF expression to prevent inappropriate vulval induction in C. elegans. Dev. Cell 10: 667–672 16678779 10.1016/j.devcel.2006.04.001

[bib5] Cunliffe V. T., 2004 Histone deacetylase 1 is required to repress Notch target gene expression during zebrafish neurogenesis and to maintain the production of motoneurones in response to hedgehog signalling. Development 131: 2983–2995 15169759 10.1242/dev.01166

[bib6] Cunliffe V. T., 2008 Eloquent silence: developmental functions of Class I histone deacetylases. Curr. Opin. Genet. Dev. 18: 404–410 18929655 10.1016/j.gde.2008.10.001 PMC2671034

[bib7] Denslow S. A. Wade P. A., 2007 The human Mi-2/NuRD complex and gene regulation. Oncogene 26: 5433–5438 17694084 10.1038/sj.onc.1210611

[bib8] Dufourcq P. Victor M. Gay F. Calvo D. Hodgkin J., 2002 Functional requirement for histone deacetylase 1 in Caenorhabditis elegans gonadogenesis. Mol. Cell. Biol. 22: 3024–3034 11940660 10.1128/MCB.22.9.3024-3034.2002 PMC133761

[bib9] Fay D. S. Yochem J., 2007 The SynMuv genes of *Caenorhabditis elegans* in vulval development and beyond. Dev. Biol. 306: 1–9 17434473 10.1016/j.ydbio.2007.03.016 PMC1955466

[bib10] Ferguson E. L. Sternberg P. W. Horvitz H. R., 1987 A genetic pathway for the specification of the vulval cell lineages of *Caenorhabditis elegans*. Nature 326: 259–267 2881214 10.1038/326259a0

[bib11] Gloghini A. Buglio D. Khaskhely N. M. Georgakis G. Orlowski R. Z., 2009 Expression of histone deacetylases in lymphoma: implication for the development of selective inhibitors. Br. J. Haematol. 147: 515–525 19775297 10.1111/j.1365-2141.2009.07887.x PMC3181219

[bib12] Greenwald, I., 2005 LIN-12/Notch signaling in *C. elegans* *WormBook*, ed. The *C. elegans* Research Community WormBook, /10.1895/wormbook.1.10.1. Available at http://www.wormbook.org

[bib13] Gupta B. P. Sternberg P. W., 2002 Tissue-specific regulation of the LIM homeobox gene lin-11 during development of the *Caenorhabditis elegans* egg-laying system. Dev. Biol. 247: 102–115 12074555 10.1006/dbio.2002.0688

[bib14] Gupta B. P. Wang M. Sternberg P. W., 2003 The *C. elegans* LIM homeobox gene lin-11 specifies multiple cell fates during vulval development. Development 130: 2589–2601 12736204 10.1242/dev.00500

[bib15] Haerty W. Artieri C. Khezri N. Singh R. S. Gupta B. P., 2008 Comparative analysis of function and interaction of transcription factors in nematodes: extensive conservation of orthology coupled to rapid sequence evolution. BMC Genomics 9: 399 18752680 10.1186/1471-2164-9-399 PMC2533025

[bib16] Hall D. H. Altun Z. F., 2008 C. elegans Atlas. Cold Spring Harbor Laboratory Press, New York

[bib17] Hanna-Rose W. Han M., 1999 COG-2, a sox domain protein necessary for establishing a functional vulval-uterine connection in *Caenorhabditis elegans*. Development 126: 169–179 9834196 10.1242/dev.126.1.169

[bib18] Hansen M. Hsu A. L. Dillin A. Kenyon C., 2005 New genes tied to endocrine, metabolic, and dietary regulation of lifespan from a *Caenorhabditis elegans* genomic RNAi screen. PLoS Genet. 1: 119–128 16103914 10.1371/journal.pgen.0010017 PMC1183531

[bib19] Hayakawa T. Nakayama J., 2011 Physiological roles of class I HDAC complex and histone demethylase. J. Biomed. Biotechnol. 2011: 129383 21049000 10.1155/2011/129383 PMC2964911

[bib20] Herman M. A. Ch’ng Q. Hettenbach S. M. Ratliff T. M. Kenyon C., 1999 EGL-27 is similar to a metastasis-associated factor and controls cell polarity and cell migration in *C. elegans*. Development 126: 1055–1064 9927605 10.1242/dev.126.5.1055

[bib21] Hsieh J. Liu J. Kostas S. A. Chang C. Sternberg P. W., 1999 The RING finger/B-box factor TAM-1 and a retinoblastoma-like protein LIN-35 modulate context-dependent gene silencing in *Caenorhabditis elegans*. Genes Dev. 13: 2958–2970 10580003 10.1101/gad.13.22.2958 PMC317160

[bib22] Hunt-Newbury R. Viveiros R. Johnsen R. Mah A. Anastas D., 2007 High-throughput in vivo analysis of gene expression in *Caenorhabditis elegans*. PLoS Biol. 5: e237 17850180 10.1371/journal.pbio.0050237 PMC1971126

[bib23] Inoue T. Sherwood D. R. Aspock G. Butler J. A. Gupta B. P., 2002 Gene expression markers for *Caenorhabditis elegans* vulval cells. Mech. Dev. 119(Suppl 1): S203–S209 14516686 10.1016/s0925-4773(03)00117-5

[bib24] Karp X. Greenwald I., 2003 Post-transcriptional regulation of the E/Daughterless ortholog HLH-2, negative feedback, and birth order bias during the AC/VU decision in *C. elegans*. Genes Dev. 17: 3100–3111 14701877 10.1101/gad.1160803 PMC305261

[bib25] Lemire B. D. Behrendt M. DeCorby A. Gaskova D., 2009 *C. elegans* longevity pathways converge to decrease mitochondrial membrane potential. Mech. Ageing Dev. 130: 461–465 19442682 10.1016/j.mad.2009.05.001

[bib26] Lu X. Horvitz H. R., 1998 lin-35 and lin-53, two genes that antagonize a *C. elegans* Ras pathway, encode proteins similar to Rb and its binding protein RbAp48. Cell 95: 981–991 9875852 10.1016/s0092-8674(00)81722-5

[bib27] Marri S. Gupta B. P., 2009 Dissection of lin-11 enhancer regions in *Caenorhabditis elegans* and other nematodes. Dev. Biol. 325: 402–411 18950616 10.1016/j.ydbio.2008.09.026

[bib28] Matus D. Q. Li X. Y. Durbin S. Agarwal D. Chi Q., 2010 In vivo identification of regulators of cell invasion across basement membranes. Sci. Signal. 3: ra35 20442418 10.1126/scisignal.2000654 PMC3917318

[bib29] Mello C. C. Kramer J. M. Stinchcomb D. Ambros V., 1991 Efficient gene transfer in *C. elegans*: extrachromosomal maintenance and integration of transforming sequences. EMBO J. 10: 3959–3970 1935914 10.1002/j.1460-2075.1991.tb04966.x PMC453137

[bib30] Newman A. P. White J. G. Sternberg P. W., 1996 Morphogenesis of the *C. elegans* hermaphrodite uterus. Development 122: 3617–3626 8951077 10.1242/dev.122.11.3617

[bib31] Newman A. P. Acton G. Z. Hartwieg E. Horvitz H. R. Sternberg P. W., 1999 The lin-11 LIM domain transcription factor is necessary for morphogenesis of *C. elegans* uterine cells. Development 126: 5319–5326 10556057 10.1242/dev.126.23.5319

[bib32] Newman A. P. Inoue T. Wang M. Sternberg P. W., 2000 The *Caenorhabditis elegans* heterochronic gene lin-29 coordinates the vulval-uterine-epidermal connections. Curr. Biol. 10: 1479–1488 11114514 10.1016/s0960-9822(00)00827-7

[bib33] Oommen K. S. Newman A. P., 2007 Co-regulation by Notch and Fos is required for cell fate specification of intermediate precursors during *C. elegans* uterine development. Development 134: 3999–4009 17942488 10.1242/dev.002741

[bib34] Penigault J. B. Felix M. A., 2011 High sensitivity of *C. elegans* vulval precursor cells to the dose of posterior Wnts. Dev. Biol. 357: 428–438 21708144 10.1016/j.ydbio.2011.06.006

[bib35] Perens E. A. Shaham S., 2005 *C. elegans daf-6* encodes a patched-related protein required for lumen formation. Dev. Cell 8: 893–906 15935778 10.1016/j.devcel.2005.03.009

[bib36] Poulin G. Dong Y. Fraser A. G. Hopper N. A. Ahringer J., 2005 Chromatin regulation and sumoylation in the inhibition of Ras-induced vulval development in *Caenorhabditis elegans*. EMBO J. 24: 2613–2623 15990876 10.1038/sj.emboj.7600726 PMC1176455

[bib37] Rimann I. Hajnal A., 2007 Regulation of anchor cell invasion and uterine cell fates by the egl-43 Evi-1 proto-oncogene in *Caenorhabditis elegans*. Dev. Biol. 308: 187–195 17573066 10.1016/j.ydbio.2007.05.023

[bib38] Schindler A. J. Sherwood D. R., 2011 The transcription factor HLH-2/E/Daughterless regulates anchor cell invasion across basement membrane in *C. elegans*. Dev. Biol. 357: 380–391 21784067 10.1016/j.ydbio.2011.07.012 PMC3164387

[bib39] Seetharaman A. Cumbo P. Bojanala N. Gupta B. P., 2010 Conserved mechanism of Wnt signaling function in the specification of vulval precursor fates in *C. elegans* and *C. briggsae*. Dev. Biol. 346: 128–139 20624381 10.1016/j.ydbio.2010.07.003

[bib40] Seydoux G. Savage C. Greenwald I., 1993 Isolation and characterization of mutations causing abnormal eversion of the vulva in *Caenorhabditis elegans*. Dev. Biol. 157: 423–436 8500652 10.1006/dbio.1993.1146

[bib41] Sharma-Kishore R. White J. G. Southgate E. Podbilewicz B., 1999 Formation of the vulva in *Caenorhabditis elegans*: a paradigm for organogenesis. Development 126: 691–699 9895317 10.1242/dev.126.4.691

[bib42] Sherwood D. R. Butler J. A. Kramer J. M. Sternberg P. W., 2005 FOS-1 promotes basement-membrane removal during anchor-cell invasion in *C. elegans*. Cell 121: 951–962 15960981 10.1016/j.cell.2005.03.031

[bib43] Solari F. Ahringer J., 2000 NURD-complex genes antagonise Ras-induced vulval development in *Caenorhabditis elegans*. Curr. Biol. 10: 223–226 10704416 10.1016/s0960-9822(00)00343-2

[bib44] Sternberg, P. W., 2005 Vulval development. *WormBook*, ed. The *C. elegans* Research Community WormBook, /10.1895/wormbook.1.6.1. Available at http://www.wormbook.org 10.1895/wormbook.1.6.1 PMC4781130 18050418

[bib45] Tang Y. Boucher J. M. Liaw L., 2012 Histone deacetylase activity selectively regulates notch-mediated smooth muscle differentiation in human vascular cells. J. Am. Heart Assoc. 1: e000901. 10.1161/JAHA.112.000901 PMC3487326 23130137

[bib46] Unhavaithaya Y. Shin T. H. Miliaras N. Lee J. Oyama T., 2002 MEP-1 and a homolog of the NURD complex component Mi-2 act together to maintain germline-soma distinctions in *C. elegans*. Cell 111: 991–1002 12507426 10.1016/s0092-8674(02)01202-3

[bib47] Verghese E. Schocken J. Jacob S. Wimer A. M. Royce R., 2011 The tailless ortholog nhr-67 functions in the development of the *C. elegans* ventral uterus. Dev. Biol. 356: 516–528 21718694 10.1016/j.ydbio.2011.06.007

[bib48] von Zelewsky T. Palladino F. Brunschwig K. Tobler H. Hajnal A., 2000 The *C. elegans* Mi-2 chromatin-remodelling proteins function in vulval cell fate determination. Development 127: 5277–5284 11076750 10.1242/dev.127.24.5277

[bib49] Wang D. Kennedy S. Conte D. Jr Kim J. K. Gabel H. W., 2005 Somatic misexpression of germline P granules and enhanced RNA interference in retinoblastoma pathway mutants. Nature 436: 593–597 16049496 10.1038/nature04010

[bib50] Whetstine J. R. Ceron J. Ladd B. Dufourcq P. Reinke V., 2005 Regulation of tissue-specific and extracellular matrix-related genes by a class I histone deacetylase. Mol. Cell 18: 483–490 15893731 10.1016/j.molcel.2005.04.006

[bib51] Wood W. B., 1988 The Nematode Caenorhabditis elegans. Cold Spring Harbor Laboratory Press, New York

[bib52] Xue Y. Wong J. Moreno G. T. Young M. K. Cote J., 1998 NURD, a novel complex with both ATP-dependent chromatin-remodeling and histone deacetylase activities. Mol. Cell 2: 851–861 9885572 10.1016/s1097-2765(00)80299-3

[bib53] Yamaguchi M. Tonou-Fujimori N. Komori A. Maeda R. Nojima Y., 2005 Histone deacetylase 1 regulates retinal neurogenesis in zebrafish by suppressing Wnt and Notch signaling pathways. Development 132: 3027–3043 15944187 10.1242/dev.01881

[bib54] Zahn T. R. MacMorris M. A. Dong W. Day R. Hutton J. C., 2001 IDA-1, a *Caenorhabditis elegans* homolog of the diabetic autoantigens IA-2 and phogrin, is expressed in peptidergic neurons in the worm. J. Comp. Neurol. 429: 127–143 11086294 10.1002/1096-9861(20000101)429:1<127::aid-cne10>3.0.co;2-h

[bib55] Zhao Z. Fang L. Chen N. Johnsen R. C. Stein L., 2005 Distinct regulatory elements mediate similar expression patterns in the excretory cell of *Caenorhabditis elegans*. J. Biol. Chem. 280: 38787–38794 16159881 10.1074/jbc.M505701200

[bib56] Zinovyeva A. Y. Graham S. M. Cloud V. J. Forrester W. C., 2006 The *C. elegans* histone deacetylase HDA-1 is required for cell migration and axon pathfinding. Dev. Biol. 289: 229–242 16313898 10.1016/j.ydbio.2005.10.033

