# Supplementary material for: Caenorhabditis elegans Histone Deacetylase hda-1 Is Required for Morphogenesis of the Vulva and LIN-12/Notch-Mediated Specification of Uterine Cell Fates
Source: G3 (Bethesda). 2013 Aug 1;3(8):1363–74. doi: 10.1534/g3.113.006999 (PMC3737176; doi:10.1534/g3.113.006999)
Supplement: Supporting Information [file supp_3_8_1363__index.html]

Caenorhabditis elegans Histone Deacetylase hda-1 Is Required for Morphogenesis of the Vulva and LIN-12/Notch-Mediated Specification of Uterine Cell Fates — Supporting Information 

# *Caenorhabditis elegans* Histone Deacetylase *hda-1* Is Required for Morphogenesis of the Vulva and LIN-12/Notch-Mediated Specification of Uterine Cell Fates

## Supporting Information for Ranawade, Cumbo, and Gupta, 2013

**Files in this Data Supplement:**

- Supporting Information - Figures S1-S2 and Table S1 (PDF, 1 MB)
- Figure S1 - A subset of genes for which RNAi-mediated knockdown caused defects in vulval morphology (PDF, 356 KB)
- Figure S2 - *hda-1::gfp* expression profile in *C. elegans* and *C. briggsae* during development (PDF, 1 MB)
- Table S1 - List of 171 genes tested by RNAi (.xls, 41 KB)
