# Supplementary material for: Caenorhabditis elegans Histone Deacetylase hda-1 Is Required for Morphogenesis of the Vulva and LIN-12/Notch-Mediated Specification of Uterine Cell Fates
Source: G3 (Bethesda). 2013 Aug 1;3(8):1363–74. doi: 10.1534/g3.113.006999 (PMC3737176; doi:10.1534/g3.113.006999)
Supplement: Supporting Information [file supp_g3.113.006999_006999SI.pdf]

G3: Genes | Genomes | Genetics

Caenorhabditis elegans histone deacetylase *hda-1* is required for morphogenesis of the vulva and LIN-12/Notch-mediated specification of uterine cell fates

Ayush Vasant Ranawade, Philip Cumbo and Bhagwati P. Gupta

Department of Biology, McMaster University, Hamilton, ON L8S 4K1 Canada.

DOI: 10.1534/g3.113.006999

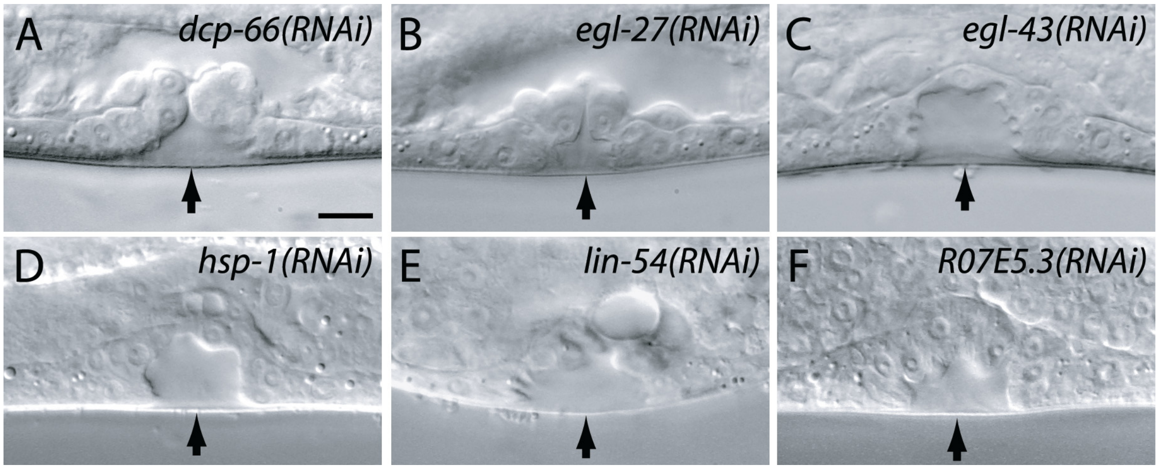

Figure S1 A subset of genes for which RNAi-mediated knockdown caused defects in vulval morphology. The phenotypes were examined in L4 stage animals. Arrows mark the center of vulval invagination. Scale bar is 10 μm.

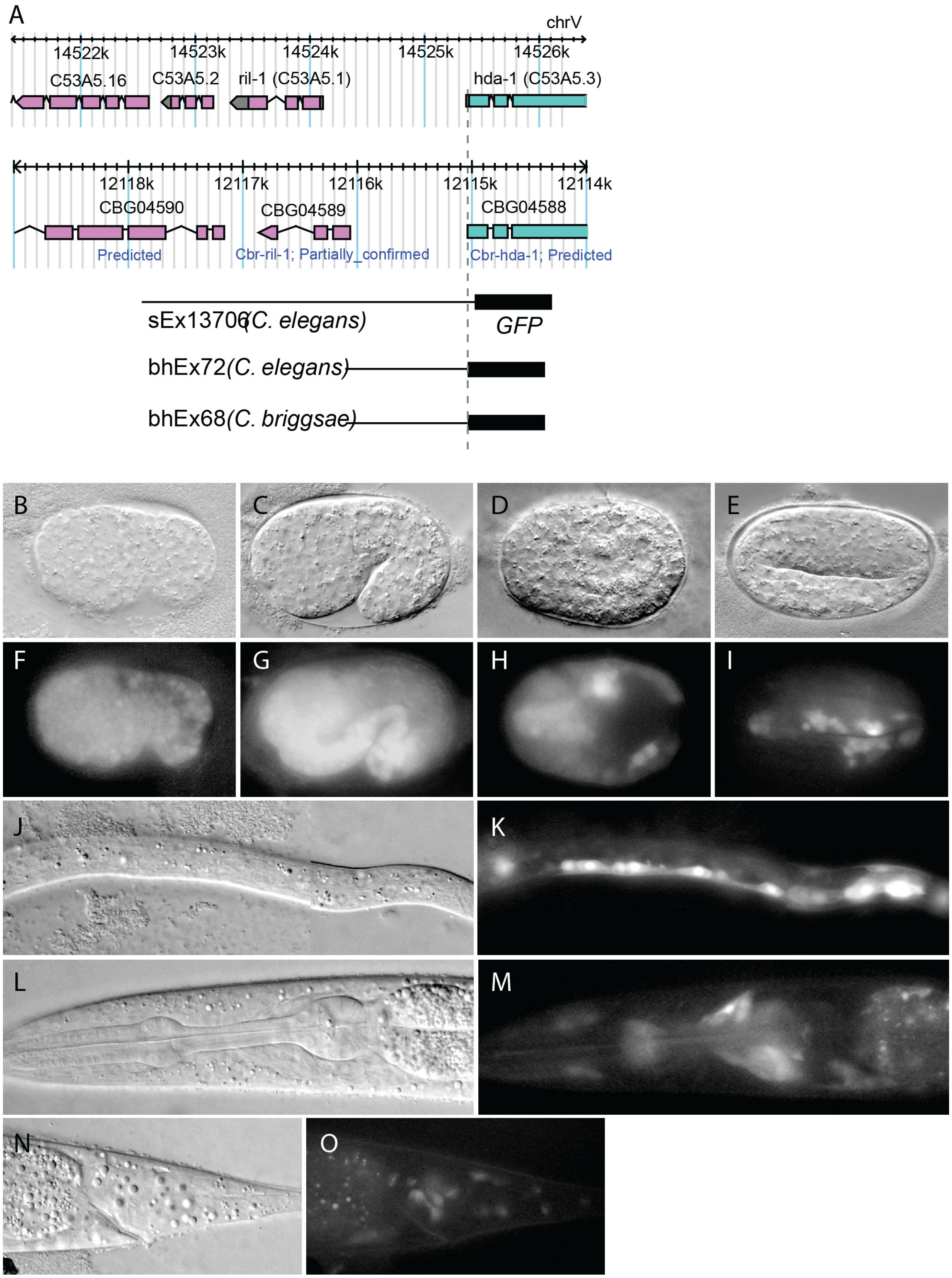

Figure S2 ***hda-1::gfp*** **expression profile in** ***C. elegans*** **and** ***C. briggsae*** **during development**
(A) Snapshots of *C. elegans* and *C. briggsae* genomic regions showing gene models of *hda-1* and upstream genes. The regions used to make the *hda-1::gfp* reporter constructs are shown. (B-O) Expression pattern of *hda-1::gfp* during development. (B, C, F, G,J-O) *C. elegans hda-1::gfp (bhEx72)*; (D, E, H, I) *Cbr-hda-1::gfp (bhEx68). hda-1* expression in embryos begins approximately 4 to 5 hrs after fertilization, towards the end of gastrulation. Expression appears to be

uniform and is observed predominantly in the head region. By the 2-fold stage, expression appears to be down regulated and confined to relatively fewer cells (J, K). In an L2 larva, bright *hda-1::gfp* fluorescence is visible in the ventral cord region. Based on their location and size, many of these cells appear to be neurons, although their exact identity is unknown. (L-O) An L4 animal shows fluorescence in the head ganglion and a subset of cells in the tail region. The anterior plane is oriented towards the left in all cases, except in E and I where it is reversed.

Table S1   List of 171 genes tested by RNAi

Table S1 is available for download at http://www.g3journal.org/lookup/suppl/doi:10.1534/g3.113.006999/-/DC1.
