# Supplementary material for: Caenorhabditis elegans Histone Deacetylase hda-1 Is Required for Morphogenesis of the Vulva and LIN-12/Notch-Mediated Specification of Uterine Cell Fates
Source: G3 (Bethesda). 2013 Aug 1;3(8):1363–74. doi: 10.1534/g3.113.006999 (PMC3737176; doi:10.1534/g3.113.006999)
Supplement: Supporting Information [file supp_g3.113.006999_FigureS1.pdf]

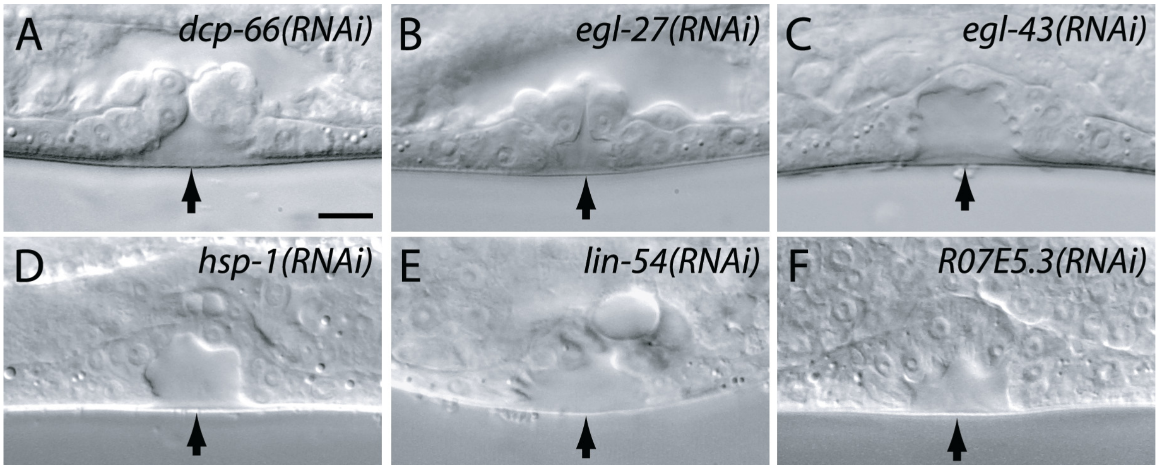

Figure S1 A subset of genes for which RNAi-mediated knockdown caused defects in vulval morphology. The phenotypes were examined in L4 stage animals. Arrows mark the center of vulval invagination. Scale bar is 10 μm.
