# Supplementary material for: Caenorhabditis elegans Histone Deacetylase hda-1 Is Required for Morphogenesis of the Vulva and LIN-12/Notch-Mediated Specification of Uterine Cell Fates
Source: G3 (Bethesda). 2013 Aug 1;3(8):1363–74. doi: 10.1534/g3.113.006999 (PMC3737176; doi:10.1534/g3.113.006999)
Supplement: Supporting Information [file supp_g3.113.006999_FigureS2.pdf]

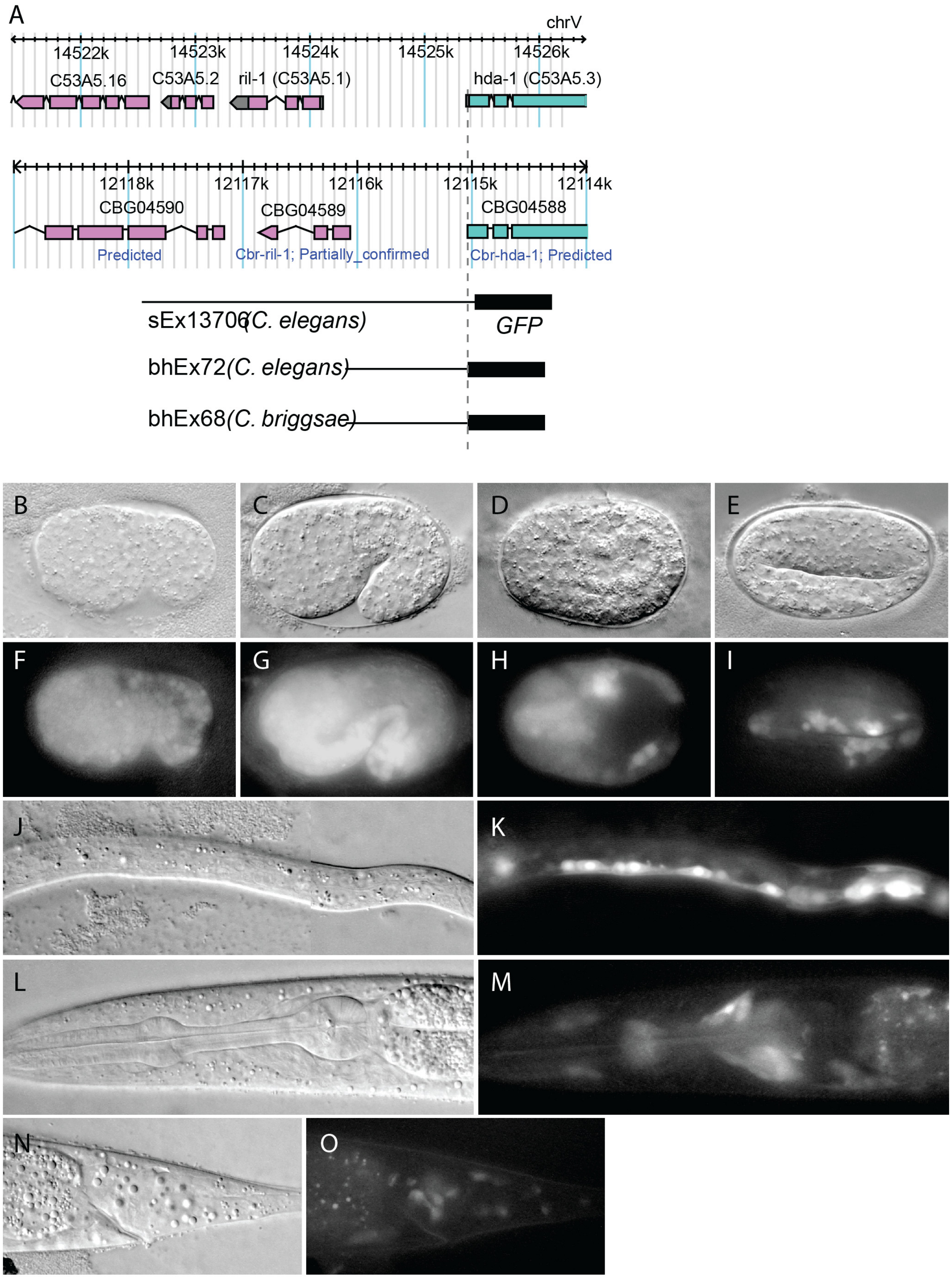

Figure S2 ***hda-1::gfp*** **expression profile in** ***C. elegans*** **and** ***C. briggsae*** **during development**

(A) Snapshots of *C. elegans* and *C. briggsae* genomic regions showing gene models of *hda-1* and upstream genes. The regions used to make the *hda-1::gfp* reporter constructs are shown. (B-O) Expression pattern of *hda-1::gfp* during development. (B, C, F, G,J-O) *C. elegans hda-1::gfp (bhEx72)*; (D, E, H, I) *Cbr-hda-1::gfp (bhEx68). hda-1* expression in embryos begins approximately 4 to 5 hrs after fertilization, towards the end of gastrulation. Expression appears to be

uniform and is observed predominantly in the head region. By the 2-fold stage, expression appears to be down regulated and confined to relatively fewer cells (J, K). In an L2 larva, bright *hda-1::gfp* fluorescence is visible in the ventral cord region. Based on their location and size, many of these cells appear to be neurons, although their exact identity is unknown. (L-O) An L4 animal shows fluorescence in the head ganglion and a subset of cells in the tail region. The anterior plane is oriented towards the left in all cases, except in E and I where it is reversed.
